# An Augmented Artificial Intelligence Approach for Chronic Diseases Prediction

**DOI:** 10.3389/fpubh.2022.860396

**Published:** 2022-03-31

**Authors:** Junaid Rashid, Saba Batool, Jungeun Kim, Muhammad Wasif Nisar, Amir Hussain, Sapna Juneja, Riti Kushwaha

**Affiliations:** ^1^Department of Computer Science and Engineering, Kongju National University, Cheonan, South Korea; ^2^Department of Computer Science, COMSATS University Islamabad, Islamabad, Pakistan; ^3^Data Science and Cyber Analytics Research Group, Edinburgh Napier University, Edinburgh, United Kingdom; ^4^Department of Computer Science, KIET Group of Institutions, Ghaziabad, India; ^5^Department of Computer Science, Bennett University, Greater Noida, India

**Keywords:** medical diagnosis, feature selection, chronic diseases, artificial neural network (ANN), prediction

## Abstract

Chronic diseases are increasing in prevalence and mortality worldwide. Early diagnosis has therefore become an important research area to enhance patient survival rates. Several research studies have reported classification approaches for specific disease prediction. In this paper, we propose a novel augmented artificial intelligence approach using an artificial neural network (ANN) with particle swarm optimization (PSO) to predict five prevalent chronic diseases including breast cancer, diabetes, heart attack, hepatitis, and kidney disease. Seven classification algorithms are compared to evaluate the proposed model's prediction performance. The ANN prediction model constructed with a PSO based feature extraction approach outperforms other state-of-the-art classification approaches when evaluated with accuracy. Our proposed approach gave the highest accuracy of 99.67%, with the PSO. However, the classification model's performance is found to depend on the attributes of data used for classification. Our results are compared with various chronic disease datasets and shown to outperform other benchmark approaches. In addition, our optimized ANN processing is shown to require less time compared to random forest (RF), deep learning and support vector machine (SVM) based methods. Our study could play a role for early diagnosis of chronic diseases in hospitals, including through development of online diagnosis systems.

## Introduction

Hospitals and online medical systems are generating a large amount of data that is beneficial for researchers to apply Artificial Intelligence (AI) techniques in building advanced models. Disease diagnosis, disease stage prediction, medical wearable, hospital stay, and death prediction are the main research areas in medical computing. Early disease detection and risk identification can motivate people to change their lousy eating, lifestyle, and exercise habits. Early chronic disease risk identification is beneficial for patients to start treatment early (1). Artificial intelligence in the diagnosis of medical diseases is a trend research area. Artificial intelligence techniques and algorithms help physicians in the decision-making process. A health specialist typically uses medical lab tests and experiments to decide whether a person is suffering from a specific disease or not. It is now easier to acquire information from previously available data using artificial intelligence techniques. In the present electronic era, massive amounts of data are generated from different health devices, namely sensors, clinical databases, social networks, and wearables (2). Except for laboratory tests and experimental results, age, gender, drug addiction, body mass index (BMI), blood pressure (BP), hypertension, lifestyle, diet, and exercise habits of patients are factors for the most common predictions of chronic diseases, including diabetes, heart attack, hepatitis, and kidney diseases (3). Artificial intelligence techniques are used to predict diseases based on available patient data. Medical diagnosis requires physicians and medical laboratories for testing, while artificial intelligence-based predictive systems are used for the early prediction of diseases.

Artificial intelligence is inspired by biological processes, especially input processing approaches used by the human brain. Artificial intelligence can learn, process inputs, identify patterns, predict, and make decisions (4). Artificial intelligence techniques are mainly divided into supervised and unsupervised learning. Supervised learning needs training on datasets available with required outputs. This training is used to build a model that is applied for future predictions (5). In unsupervised learning, similar data points are combined into groups called clusters. Each cluster consists of data points with similar characteristics (6). Supervised learning is further divided into two categories: Classification and Regression. Classification is used to predict the final class labels with positive and negative results. Classification algorithms are used to build a predictive model based on historical data and indicate classes of unlabeled data.

According to the World Health Organization (WHO) analysis, 41 million people die from chronic diseases, which is 71% of total deaths each year. Chronic diseases include heart attacks, cancer, diabetes, kidney disease, liver disease, etc. Diabetes killed 1.6 million people in 2016 (7). This rapid growth of diabetes patients requires an early detection system to treat their disease early. Diabetes mellitus occurs with an increase in blood glucose levels. There are mainly two types of diabetes: Type I diabetes occurs when the pancreas stops producing the required insulin. Type II diabetes occurs when a patient's body is not responding (8). Diabetes is a lifelong disease that affects different body organs, including the kidneys, heart, and eyes. Heart diseases are considered as another main reason for death in middle and old age. According to cardiovascular statistics provided by the World Health Organization (WHO), more than 17.9 million deaths occur due to heart stroke (9). Physicians refer to medical tests for heart disease diagnosis like electrocardiogram (ETC), echocardiogram (ECG), computerized tomography (CT scan), etc. In another way, early heart attack symptoms are used to predict disease so that the clinical burden can be reduced and physicians or patients themselves can diagnose their heart disease. Family history, blood pressure level, cholesterol, stress level, eating habits, age, weight, smoking, and alcohol consumption are the main factors that are examined to predict heart disease before occurrence.

Conventional methods of kidney disease diagnosis involve ultrasound, urine or blood tests, etc., which are followed by kidney dialysis, transplant, or simple medication. Almost one out of three diabetes patients suffer from kidney failure (10). Kidney diseases are predicted early by using automatic prediction systems. Early detection may reduce the mortality rate and cost associated with lab experiments. Kidney diseases are related to bad diet habits and unhealthy living; these attributes are easily used to build a predictive model. Hepatitis is an inflammation in the liver due to viral infections. It is another rapidly spreading chronic disease with more than 350 million patients (11). The reason behind death is hepatitis shows symptoms like viral infections, namely, fever, fatigue, and liver infections, etc.

Artificial intelligence makes it possible to diagnose Hepatitis C early without clinical expense (12). Breast cancer is overgrowing in European and Asian countries. It has become the most frequent type of cancer, causing the most incredible death rate among women that is 15% (13). Breast cancer diagnosis involves two steps: detection and screening. It is crucial to predict breast cancer early to provide necessary medical services and care. Many researchers contribute to the detection and prevention of breast cancer at an early stage. Breast cancer became the second leading cancer that causes the most deaths among women. It is necessary to propose a predictive analysis model for cancer diagnosis in its early stages. Early diagnosis of breast cancer will lead to early treatment and may reduce the risks of death. Web-based systems can also reduce the cost of lab experiments; therefore, they are beneficial to the patient and medical staff as well. Improvements in medical database systems help to generate a large amount of data that is unable to handle by a human. Artificial intelligence is used to extract patterns in huge datasets and to find the labels of data for patient disease diagnosis (14).

However, health-related data are more sensitive; therefore, they must be handled carefully to generate accurate results. Real-World data has many issues, including noisy data, class imbalance, missing values, and irrelevant entries (15). Different data pre-processing approaches have been applied in previous research to increase the performance of medical diagnosis systems (16). Data pre-processing plays a vital role in extracting valuable features in data. Data pre-processing consists of extraction, cleaning, and transformation steps (14). Chronic diseases are affected by hypertension, gender, obesity, age factors, smoking, unhealthy diet, and lifestyle; therefore, it is difficult to identify the disease from all functional aspects.

Furthermore, different hospitals or medical diagnostic laboratories have the attributes of other patients with the same strategies. A preferable method to handle other characteristics in the dataset is feature selection. Feature selection approaches make it possible to find weights of different factors so that only the most influencing factors are used for detection. Feature selection is used to select the most significant features and to eliminate irrelevant features from the dataset. Feature reduction techniques reduce processing time and increase classification performance that is affected by useless features in the data (17).

Some datasets are available online in different data repositories for research purposes. To find and evaluate the symptoms of other diseases, the researchers applied various algorithms and built different effective prediction models. These models are applicable for the selected dataset to diagnose any specific disease early. In comparison, many research studies have proposed methods to predict various diseases, e.g., Diabetes, Cancer, Parkinson's, Heart Attack, Depression, Hypertension, etc. However, the medical diagnosis systems focus on a specific disease with limited attributes. Previous studies have shown that traditional methods for predicting chronic diseases are inadequate due to very limited feature selection techniques. Therefore, significant features identification and machine learning techniques are important to improve the performance of chronic diseases. The main contributions of this research paper are as follows.

- The significant features are chosen to eliminate redundant and inconsistent data through particle swarm optimization, which improves the efficiency of prediction model.
- An artificial neural network predicts five chronic diseases, diabetes, breast cancer, hepatitis, heart, and kidney.
- The k-fold cross-validation is used to train and test the proposed approach. Classification results are compared to a decision tree, random forest, deep learning, KNN, Naive Bayes, SVM, and logistic regression on chronic diseases datasets.
- The experimental results show that the proposed approach achieved a 99.76% accuracy and predicts chronic diseases more effectively and efficiently than other state-of-the-art models.

The rest of the study is arranged as follows: Section Literature Review represents a comprehensive review of studies in the previous literature. Section Materials & Methods discusses the materials and methods. Section Results and Discussions presents experimental results and comparative analysis. Finally, Section Conclusion concludes, and section Future Work and Limitations provides future directions and limitations for the research.

## Literature Review

The literature review provides a clear view of previous approaches and potential areas. The latest literature reviews different disease detection methods using artificial intelligence approaches with or without other feature selection methods.

A research study proposed a machine learning-based predictive model to diagnose three primary chronic diseases, including Diabetes, Kidney, and heart diseases. The feature selection technique based on adaptive probabilistic divergence is used to select the most valuable features. The study concluded that the proposed approach gave the highest accuracy by optimizing the most significant features for disease detection (2). A feature selection-based machine learning algorithm is proposed to predict three chronic diseases, namely, diabetes, heart attack, and cancer. The incremental feature selection approach with Convolutional Neural Network (CNN) is applied to anticipate disease presence. The proposed method showed 93% classification accuracy in less computation time (18). Another study is conducted to explore the essential features of often chronic diseases. Feature selection approaches, including Information Gain, Gain Ratio, and correlation-based approaches, are applied. Several subsets of top-ranked features are then used in building the Random Forest prediction model. It showed that exploring the most significant features is significant for the medical diagnosis process (19). In another research study for chronic disease prediction (20), the Stacked Generalization approach is used to enhance the performance of classification algorithms. Five classification algorithms are compared: Decision Tree (DT), k-NN, SVM, Logistic Regression (LR), and Naive Bayes, to outperform five chronic disease prediction models. It is found that the Stacked Ensemble approach enhances the model performance and achieves the highest accuracy of 90%. Diabetes, Breast Cancer & Kidney diseases are predicting in (21), using classification algorithms. Rough K-means clustering is used to explore and eliminate ambiguous objects in datasets. It is observed that classification model applied with extracted features gave improved results compared to conventional models.

Early breast cancer prediction is an emerging research area in artificial intelligence. Many studies are conducted for breast cancer prediction at an early stage using online techniques and prediction models. The fuzzy rule-based classification is used for classification purposes, while fuzzy temporal rules are used to determine highly contributing factors for online breast cancer prediction. The results show that feature selection and fuzzy rule-based classification improve the accuracy of classifiers (22). Another study focused on feature filtering techniques to predict breast cancer early. Frequent item-set mining is used to select the essential features in patients' datasets. The decision tree, Naive Bayes (NB), k-Nearest Neighbors (k-NN), and Support Vector Machine (SVM) are compared, and it is found that SVM outperforms other models (23). A research paper focused on reducing erroneous prediction results that is false positive and false negative. Information gain with the Genetic Algorithm approach is utilized to rank highly significant features in the dataset. Classification of positive and negative results is done by SVM. This approach applied to two independent datasets increased the prediction accuracy and reduced prediction cost (24). In another research study (25), five classification models, namely SVM, K-NN, ANN, random forest, and logistic regression, are applied to predict early breast cancer. In this study, the Pearson correlation is applied to extract the most influencing features, and after feature selection, the ANN model obtained the highest accuracy of 98%. Two breast cancer datasets are used in (26), to differentiate cancer patients from healthy people. A genetic algorithm is used to select the most significant features in datasets.

Three classification algorithms, multilayer perceptron (MLP), probabilistic neural network (PNN) and radial based function (RBF) are used for the classification of breast cancer (27). MLP requires more processing time for the training model and assigning weights to its neurons than RBF and PNN. The highest accuracies of 97%, 98%, and 100% are achieved with MLP, RBF, and PNN. Another research study (28) applies SVM with multiple kernels to predict breast cancer. An energy-based shape histogram is used to extract the most significant features. The proposed model achieved the highest accuracy of 99%.

Diabetes prediction at an early stage can lead to saving patients' life. A research paper compares classification methods like Artificial Neural Network (ANN) and Random Forest with clustering technique k-means clustering. Principal Component Analysis (PCA) is used to select significant components. The study proved that PCA increases the accuracy of the prediction of diabetes, while body mass index (BMI) and glucose level strongly correlate with diabetes (28). Prolonged diabetes can cause vision loss, which is called diabetes retinopathy. A research study is conducted to predict the side effects of diabetes on eyesight. K-NN, Decision Tree, Multilayer Perceptron, and SVM are used to predict feature selection. The number of accurate predictions is compared before and after feature selection, demonstrating that feature selection approaches increased accuracy and sensitivity (29). Some techniques are used for diabetic retinopathy using PCA (30) and deep neural network (31). In (32), BMI is predicted from the images using the computer vison and machine learning technique. Federated learning has a wide application in big data (33). The probabilistic procedure with sensors is used for censorious procedures (34). Some patients have diabetes with hypertension; therefore, a research study is conducted to predict diabetes type II and hypertension in both individuals. Synthetic minority oversampling is used to solve data distribution problems and the ensemble method is used to predict hypertension and type II diabetes. This study showed that pre-processing of data prior to model building enhances prediction accuracy (35). Machine learning methods are also used for the detection of some other medical diseases detection (36–38), text mining (39–42) and network security (43–47). Another analysis is conducted to predict the risks associated with diabetes. Logistic regression, Decision Tree, ID3, C4.5, k-NN, and Naive Bayes are used for classification. Irrelevant features are detected and reduced using PCA and PSO algorithms. A comparison of both feature selection techniques is performed in terms of improved accuracy and processing time. It shows that feature reduction is a powerful technique to enhance predictive model accuracy (48).

Iris-based and physiological features are used to predict diabetes type II, using decision trees and SVM. Some methods used the fuzzy for ergonomics-related disorders (49), Convolutional Neural Networks for Syndrome (50), object detection (51) and gender classification (52). Three ensemble techniques, AdaBoost, Bagging, and K-NN, are also used with Principal Component Analysis (PCA) to enhance classification performance. The highest accuracy of 95.81% is obtained with the most significant features (53). A convolution neural network (CNN) is a feed-forward and feature extractor Neural Network. In a study (54), early diagnosis of Diabetes Type II is performed by using CNN. A dataset of steel mill workers, including demographics, physical activities, and hypertension features, is used to implement CNN. The highest accuracy of 94.5% is obtained to diagnose diabetes patients from all workers' datasets. Diabetes patients are usually affected by some other chronic diseases. A research study (8) highlighted the features most common in heart and diabetes patients. Heart disease is found to be predicted early in diabetes patients by applying classification and regression techniques.

In recent decades, heart attacks have been one of the leading causes of death. Random forest is used in conjunction with a linear model to predict heart disease based on patient data in a study. The most significant features are classified using an a priori method, which showed an increase in accuracy. When compared to existing classification techniques, the suggested ensemble model produces the best results (55). Optimization techniques are used to deal with complex data by reducing irrelevant and functional data attributes. In a study, the Particle Swarm Optimization (PSO) and Ant Colony Optimization (ACO) algorithms are combined for redundant feature reduction. This approach is applied with different classification algorithms and is evaluated using F-measure, accuracy, precision, and recall. Feature optimization gave the highest accuracy of 99.65%, which is a remarkable achievement (56). Software quality (57–59) and semantic methods (60, 61) are also used in algorithm evaluations. A hybrid approach combines different AI techniques and evaluates them as a single algorithm. A research study applied various AI algorithms for heart disease prediction to find the best classifier. The best performing algorithms are then combined, and the results of the hybrid approach are compared with the results of individual techniques. The proposed hybrid algorithm gave more accurate results as all algorithms functions are combined to calculate a majority vote for a positive class. A drawback of using a hybrid approach is that more processing time is required (62). Another study applied a Fuzzy Rule-based classification model for heart attack risk prediction. Rough set theory identifies the most influencing features, and features that do not affect prediction results are eliminated. The Genetic Algorithm is used to reduce time complexity and optimize prediction results. Research proved that the proposed model is efficient with a large number of features (63, 64). A comparative analysis is presented in (65) for the diagnosis of cardiovascular disease. The experimental setup comprises classification algorithms, namely Logistic Regression, Decision Tree, and SVM. Different subsets of features are used to evaluate classification results. The highest accuracy of 82.97% is achieved by using a decision tree with forward and backward selection approaches.

A study is proposed to predict whether a patient would survive or die because of hepatitis. This study implemented SVM with multilayer perceptron for the binary classification of patients. Principal component analysis (PCA), a feature selection approach, is applied before prediction and three factors of zero correlation with the predictive class are eliminated. The proposed hybrid algorithm gave a higher accuracy than state-of-the-art classifiers (66). A combination of several prediction techniques results better than an individual model. Another study suggested a model for hepatitis diagnosis using ensemble learning techniques. A Neuro-fuzzy system is used for patients' diagnosis with dimensionality reduction algorithms. The results demonstrate that the ensemble neuro-fuzzy model performed more accurately than ANN (67). A study is conducted to highlight risk prediction factors for hepatitis C using a random forest classifier. Chi-square and *P*-value statistical functions are used to estimate the importance of various risk determining factors. The importance of the factor is calculated several times and each important timeless factor is eliminated until the required predictive model has 98.3% accuracy. The research found that gender, drug addiction, frequent hospitalization, and migration from the area with a high HIV occurrence rate are the essential factors for hepatitis diagnosis (68). A two-step feature transformation method is proposed to increase the accuracy of the hepatitis prediction model. At first, the most valuable features are ranked based on their significance, and after feature reduction, the attribute space is expanded. This hybrid method manifested improved prediction results achieved in less computation time (11). Esophageal variants occur most commonly due to the side effects of hepatitis. The esophageal prediction model is proposed in a research study, with six feature selection approaches. Information Gain, PCA, and correlation are used to select the most significant features. The Greedy Approach, PSO, and Genetic Algorithm are applied to create subsets of the dataset by exploring the correlation between different features. This study concluded that Naive Bayes outperformed compared to ANN, Decision Tree, Random Forest, and SVM (12). Some other machine learning and soft computing methods are mainly used for the chronic disease detection (21, 69–71).

Ant Colony Optimization is used for reducing irrelevant or least significant features. The study presented that ACO feature selection enhances the performance of supervised learning classifiers (17). Kidney disease stage identification is performed by applying artificial intelligence techniques to various patient attributes. The most influencing features in the prediction of kidney disease stage are serum creatinine, urine, and albumin. Probabilistic Neural Network beat other prediction models with 96.7% accuracy (72). Random Forest is applied to predict chronic kidney disease in patient data. Dataset is divided into demographics and clinical data, including patients' age, BMI, gender, diabetes history, hypertension, etc. Prediction results are estimated by R2 (73). Data are preprocessed by outlier detection, normalization, and irrelevant data features are reduced. The random subspace outperformed with 100% accurate results and proved that ensemble approaches are better to use than individual classifiers (74). Besides kidney disease presence prediction, kidney transplantation survival is another critical aspect of prediction. The study proposed a prediction model for patients' survival after kidney transplants. Information Gain and Naive Bayes are used for the most significant feature selection. When evaluated with Accuracy and F-measure, K-NN gave higher accuracy than other classifiers (75). Another study classifies kidney patient and healthy people data by applying correlation-based feature selection approaches. Three feature ranking approaches, namely ReliefF, information gain, and gain ratio, explore the most significant attributes (76). Table 1 shows the summary of previous literature that presents their problem statements, diseases to be predicted, and proposed solutions.

**Table 1:** Summary of the literature review.

**Year**	**Problem statement**	**Diseases**	**Proposed solution**	**Reference**
2021	Disease prediction with the features using machine learning	Diabetes and breast cancer	SVM	(77)
2020	Different feature selection approaches are compared to evaluate their recall, precision, and F1 measure performance.	Diabetes, Kidney and Heart Attack	Adaptive probabilistic divergence is used to select most useful features.	(2)
2020	To predict the presence of three chronic diseases.	Diabetes, Heart attack, and cancer	Incremental Feature Selection Approach with Convolutional Neural Network (CNN)	(18)
2019	To explore most important features for different chronic diseases.	Heart, Hepatitis, Diabetes, Cancer	Including information gain, gain ratio, and correlation-based approaches.	(19)
2020	Enhance the accuracy of a prediction model	Chronic Diseases	Stacked Ensemble approach	(20)
2020	To enhance the classification results using the clustering method	Diabetes, Cancer & Kidney diseases	Rough K-means clustering	(21)
2019	Focused on feature filtering techniques to predict cancer in an early stage.	Cancer	Decision Tree, Naive Bayes, k-Nearest Neighbors, and Support Vector Machine	(23)
2020	Extract the most influencing features	Cancer	Pearson correlation with ANN	(25)
2019	Enhance classification performance using significant attributes	Diabetes	A hybrid of AdaBoost, Bagging, and K-NN	(53)
2019	To predict diabetes using demographics and hypertension data	Diabetes	convolution neural network (CNN)	(54)
2020	To highlight features most common in heart and diabetes patients.	Diabetes and Heart Disease	Supervised learning classification and regression	(8)
2019	Heart disease prediction based on patients' data.	Heart Attack	Random Forest	(55)
2019	Feature reduction in heart patients' data.	Heart Attack	Particle Swarm Optimization (PSO) and Ant Colony Optimization (ACO)	(56)
2020	To explore the most significant features	Hepatitis	Principal Component Analysis (PCA)	(66)
2019	Highlight hepatitis C risk predicting factors	Hepatitis	Random Forest	(68)
2020	Feature transformation is used to increase the accuracy of the hepatitis prediction model	Hepatitis	Classification	(11)
2019	Irrelevant significant features reduction for kidney disease prediction model	Kidney Failure	Ant Colony Optimization	(17)
2019	to predict chronic kidney disease based on demographic data.	Kidney Diseases	Random Forest	(73)
2020	Ensemble techniques for kidney disease prediction	Kidney Diseases	Decision Tree, k-NN and Naive Bayes	(74)
2019	A prediction model for patients' survival after kidney transplant	Kidney Diseases	Information Gain and Naive Bayes	(75)

## Materials and Methods

**Figure 1:**
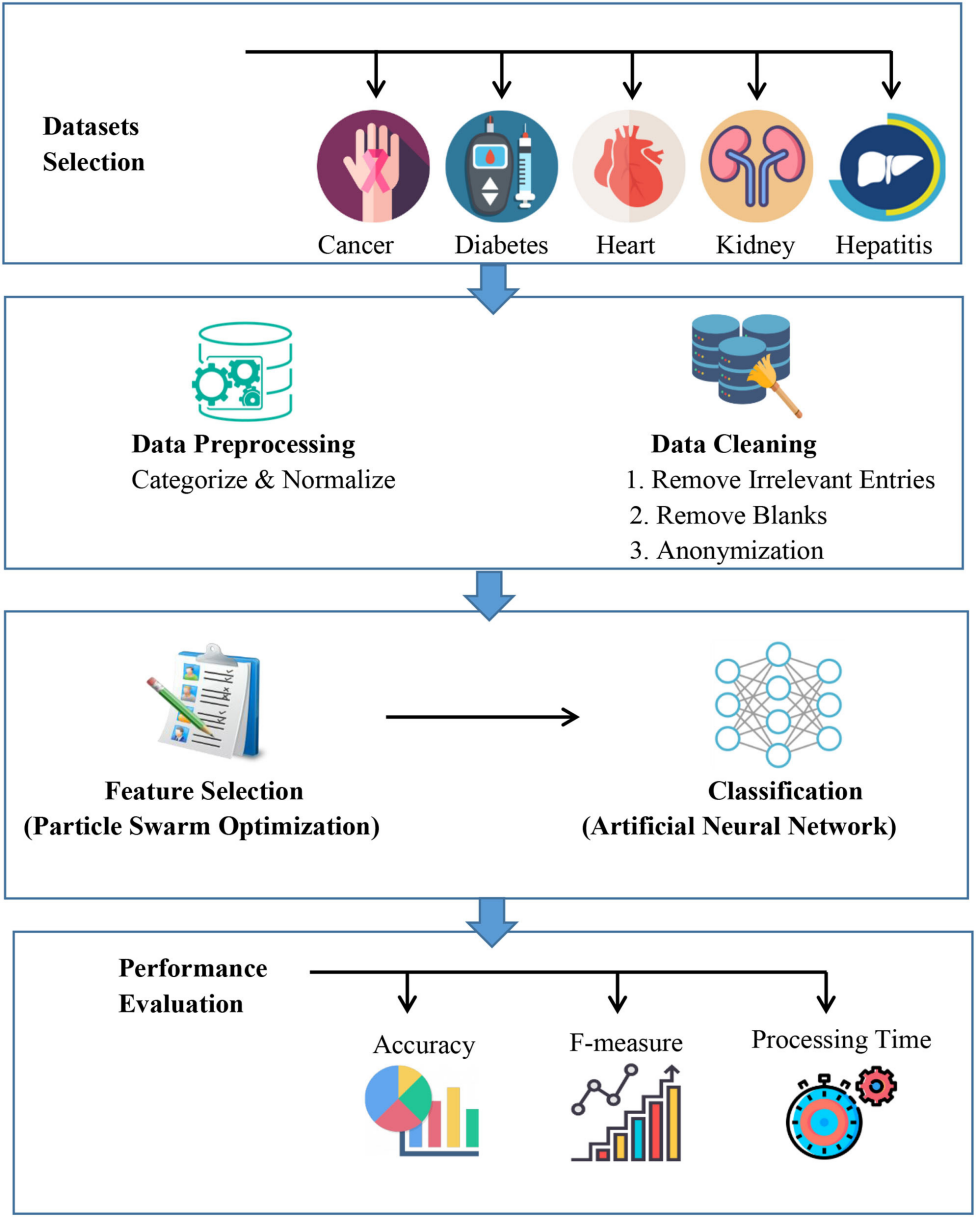
Proposed approach.

In this section dataset selection, data pre-processing, feature selection, proposed model architecture, classification methods, performance evaluation, experimental tools and setup and validation method are discussed. Figure 1 describes the proposed automated artificial intelligence approach. The datasets are selected, and pre-processing is applied. The features are extracted with particle swarm optimization and then an artificial neural network is utilized.

### Dataset Selection

Datasets for five chronic diseases, including Breast Cancer, Diabetes, Heart, Hepatitis, and kidney disease, are collected from multiple online sources e.g., Kaggle, Dataworld, Github & UCI machine learning repository. Four datasets are previously used in a research study (19), to predict different diseases, while other datasets are used in multiple research studies. Each dataset has information about numerous patients with various diseases; therefore, dataset features and instances vary. All datasets used in this research study have numeric and categorical data features based on patients' demographics and medical test results. Breast Cancer data attributes include the characteristics extracted from digital images of fine-needle aspirates of different breast tissues. Diabetes datasets are extracted from patients' demographic profiles and medical test results, including gender, age, glucose level, blood pressure, and body mass index, etc. Two datasets containing heart disease symptoms and patients' data are used to detect a person's heart disease or not. Heart disease dataset attributes used for detection include demographics, chest pain, cholesterol level, glucose level, ECG results, maximum heart rate, depression, and exercise-induced angina. For the early prediction of hepatitis, demographics and medical lab test attributes are used. Dataset features used for diagnosis are categorical, including age, gender, antivirals, liver size, fatigue, discomfort, bilirubin, albumin, etc.

The kidney disease dataset is used to diagnose kidney disease presence, using features like sugar level, red and white blood cell count, glucose level, blood urea, diabetes presence, and hunger. All above datasets consist of multiple features; however, not all features are equally significant for disease diagnosis. This research study finds that feature selection approaches help to optimize classification results by considering useful features only. Therefore, for each disease, two publicly available datasets are used to verify the classification results. However, for kidney disease classification, only one dataset is found on multiple online platforms. Table 2 describes the dataset attributes and their statistics.

**Table 2:** Datasets used for classification.

**Diseases**	**Dataset name**		**No. of attributes**	**Total** **entries**	**No. of positive results**	**No. of negative results**	**References**
Cancer	Breast Cancer Wisconsin (Diagnostic) Data Set (Dataset 1)		31	569	212	357	(78)
Cancer	Breast Cancer Wisconsin (Dataset 2)		10	699	240	459	(79)
Diabetes	Pima Indians Diabetes Database (Dataset 1)		9	768	268	500	(80)
Diabetes	Diabetes Classification (Dataset 2)		15	390	60	330	(81)
Heart Attack	Heart Disease UCI (Dataset 1)		14	303	138	165	(82)
Heart Attack	Heart Disease Prediction (Dataset 2)		13	270	150	120	(83)
Hepatitis	Hepatitis (Dataset 1)		19	142	80	62	(84)
Hepatitis	Indian Liver Patient Records (Dataset 2)		11	584	168	416	(85)
Kidney	Kidney Disease Dataset (Dataset 1)		26	189	74	115	(86)

### Data Pre-processing

In predictive analytics, data quality is a significant factor, low quality of data leads to incorrect results. Due to their varied uses and sources, real-world medical data sets have many outliers, missing values, and irrelevant features. To make the artificial intelligence algorithm more productive, pre-processing is essential before using data for model training (87). Data pre-processing is a conventional artificial intelligence technique to convert raw data into useful information. Data pre-processing involves various steps needed to obtain relevant and valuable data from the original dataset. Data pre-processing steps involve data extraction, anonymization, integration, cleaning, outlier detection, and duplicate removal (15). Therefore, all the datasets are pre-processed before using data for prediction. In these datasets, it is observed that the patient's glucose level, BMI, skin thickness, insulin, blood pressure, and age have a minimum value of zero, which is impossible. Such outliers are replaced with average values. Some instances have blank entries, which are removed by using the replace missing values operator to make an accurate detection. Table 2 presents the datasets and their values.

### Feature Selection

Many datasets for all diseases are available in the hospital's database systems and online repositories. These datasets have different features for multiple usages; not all available features contribute to specific disease detection. Data pre-processing is an essential step before applying a model for the classification of data. The model trained on a dataset with wrong entries may lead to misleading results. Similarly, not all attributes in a dataset contribute to the detection process equally. Including irrelevant features increases the processing time of the model and it also decreases model performance. If all attributes are used for predictive analysis, classifier performance is affected negatively. For hospitals and medical labs, it is dependent on the physician's experience to diagnose a disease from different symptom input values. However, different feature selection approaches allow examining the contribution of various features on results. The main functionality of feature selection techniques is used to analyse that how each attribute takes part in output prediction. Feature selection is an essential approach to be used before model construction to redue data complexity by eliminating irrelevant and useless features from data. Feature selection approaches reduce the data size so that the model takes less time in the training and testing phases. Feature selection is beneficial as it reduces the data size so that processing time, space, and power consumption are also reduced (15). With a fewer number of features, the classification model becomes more interpretable and yields more effective results. Features with zero or very low contributions are eliminated by using feature selection. There are various feature selection methods. Information Gain (IG) is one of the most effective methods for feature selection. Information Gain is measured by entropy, which is the uncertainty of being selected. A low entropy value represents more chances of being selected as a final class label (16). Information Gain calculates the weight of each attribute to estimate its contribution to the last class selection. The highest weight represents the feature that has the most information about final class selection, therefore considered as the most influencing feature.

We present the significance of selecting the most significant features using Particle Swarm Optimization (PSO). PSO results are compared with Information Gain to evaluate its performance. Particle Swarm Optimization (PSO) is another feature ranking technique. PSO is introduced in 1995, inspired by groups of birds and fishes searching for food in a swarm group. Each species in the swarm searches for food, and the whole group follows that individual nearest to the food to travel in the right direction in minimum time. Similarly, the PSO technique is used to find an optimal solution for a problem consisting of various features (48). PSO is an evolutionary algorithm developed on the social behaviors and movements of species. PSO is applied to optimize classification results by assigning weights to the most significant features based on their previous results (88). Thus, only the significant features are used for the prediction process to decrease processing time and to enhance the efficiency of prediction model. The ANN classification model based on the most significant features obtained by PSO. Using PSO, all attributes are assigned weights according to their contribution to the classification process. In addition, a baseline information gain approach is also used to compare its results with the proposed model.

### Proposed Model Architecture

The proposed model architecture is described in this section. Artificial intelligence algorithms are used to construct the chronic disease detection model. Artificial intelligence is an advanced machine learning approach to build detection models that can receive input data, train a model, and predict the output of future data. These model-building techniques are divided into two main categories: supervised learning and unsupervised learning. Supervised learning models are provided with a set of input data with desired output labels to construct a predictive technique for future datasets. Supervised learning algorithms include a set of tree-based, instance-based, probability-based, and ensemble algorithms (89). Classification is a supervised machine learning approach that works under supervised learning to predict the final class. In classification, the output variable is nominal such as patients' disease diagnosis that is positive or negative (5).

**Figure 2:**
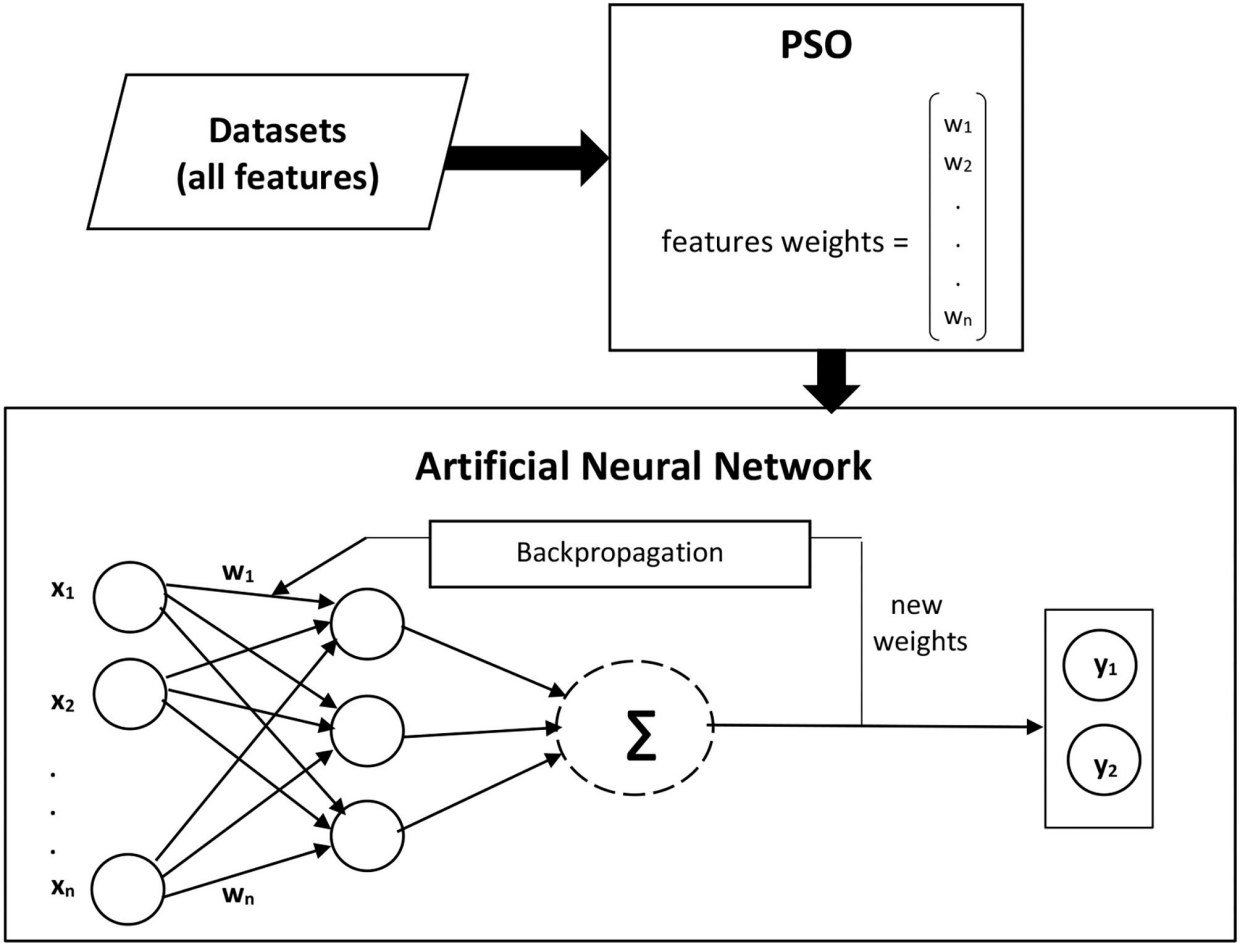
Proposed model architecture.

The forward and backpropagation are evaluated during the ANN learning phase, and it is observed that forward propagation gives higher accuracy. Therefore, an Artificial neural network with forwarding selection is used to predict disease presence. Dataset features with and without feature selection approaches are given as an input to the first layer. The processed output of the first layer is fed into the second layer as input until it reaches the output layer. Cross-validation with tens folds is used for the training and testing process. Each dataset is divided into 10 folds, and nine folds are used for training and one-fold for the testing process in such a way that each fold must be used for testing. The different number of training cycles is applied to get the best classification results, and finally, ANN with 200 training cycles is used to finalize the prediction results. ANN learning rate controls how fast Neural Network will learn a prediction problem. It is a configurable hyper-parameter tuned during the ANN training process. A learning rate of 0.01 is used, as a lower learning rate allows Neural Network to learn more optimally. For the breast cancer diagnosis, 31 input features are fed to the proposed model. After classification of the first breast cancer Wisconsin (diagnostic) dataset, the second breast cancer Wisconsin dataset with 10 features is provided as an input to the proposed model, and the results are recorded. Similarly, for the other four diseases detection, datasets with different features, as described in Table 2, are fed into the proposed model, and results for each disease diagnosis are recorded. For classification, at first, ANN is trained with all available features to classify patients and healthy people in each dataset. In the second step, features are assigned weights using Information Gain, and classification is made based on the weighted features. In the third step, PSO is applied to each dataset, and its results are compared with classification performance without feature selection and with Information Gain. Figure 2 presents the architecture of the proposed model, where w stands for the weights assigned to the features, while x and y are used for input and output, respectively.

### Classification Algorithms

The Artificial neural network classification algorithm is compared with seven state-of-the-art classification algorithms, namely, Naive Bayes, K-NN, Decision Tree, Random Forest, Logistic Regression, SVM, and Deep Learning. There are various classification algorithms to find a correlation between patients' attributes and disease symptoms, but all algorithms have some limitations in input requirements, model construction function, and required computation time (15). ANN is used for five different chronic disease detection. It is inspired by biological neural networks that pass information from the human brain to other body parts by passing through neurons. ANN is a classification algorithm that learns from training examples and applies the trained model to future datasets to predict their output (16). ANN is a set of connected neurons comprising input, processing, and output layers. Each neuron is connected to its adjacent neurons (90), whereas each connection is assigned some weight. These weights are adjusted in the training phase to weaken or strengthen the relationship based on its contribution toward output prediction. Model training is done through backward or forward propagation (91). Naive Bayes is suitable for both numerical and nominal data with multiple dimensions. Naive Bayes is the Bayesian theorem-based classification algorithm, which works on conditional probability, the class with high probability is considered as the final output. Naive means each feature in the dataset is independent of other features and their presence does not affect others. Naive Bayes can be applied to large datasets as its feature independence makes classification faster than other classification algorithms (16).

KNN is the simplest and easiest algorithm used for classification. K is the nearest neighbor of a data sample whose class is being predicted (91). KNN, also referred to as lazy learning (15), allocates weights to the contributing neighbors so that the nearest neighbor will have more influence on prediction. Most commonly, Euclidean distance is used to calculate the distance of all neighbors. KNN assumes that similar instances occur close to each other; hence, the nearest neighbors contribute to predicting the final class (75). KNN with a weighted vote is used for classification in this research study, with *k* = 10 which represents 10 nearest neighbors. A Decision Tree is the most straightforward classification algorithm, which follows a tree-like structure starting from the root node and reaches the output node (leaf) by evaluating some conditions. Each node is connected to its next node by a connection (branch); this connection represents a condition, if true, then reaches the next node. Since the decision tree works well with categorical data, it is most suitable for the dataset with fewer features (5). This paper uses decision trees with a maximum depth of 10 nodes for predictive analysis. Numerical data is changed to categorical data by using numerical with the nominal operator. Feature selection techniques are applied to select the most significant nodes so that the classification becomes more effective with a smaller number of features.

Random Forest is an ensemble learning technique used for prediction. Random Forest is a more effective algorithm than a decision tree as it combines a set of multiple decision trees by selecting random data samples and predicts the final class based on the majority vote. More trees mean more robust prediction, but implementing many trees requires more processing time (16). The primary aim of ensemble techniques is to strengthen weak algorithms to build a robust predictive model. In this paper, Random Forest with 100 trees is constructed. Logistic regression is a supervised learning classification technique used to output binary classes. It classifies the output by using probability function, results higher than 0.5 are classified as positive while <0.5 are predicted as negative. It operates numerical input data; therefore, categorical attributes are changed to numerical by using nominal to numerical operators (15). SVM is used to classify linear as well as multidimensional data. In SVM, a hyperplane is used to separate different classes of variables. Both sides of the hyperplane represent two other classes. The Euclidean function is used to calculate the distance between variables so that the hyperplane is drawn in its best place (12). SVM is used for both classification and regression problems. In this paper, SVM with the kernel (dot) is implemented to classify data into binary classes. Before the model, nominal building data is converted to numerical. Deep Learning (DL) is a machine learning approach (92), comprised of a Neural Network with multiple hidden layers. The label with the highest value is finalized as output class (93). Since more training gives more accurate results, but it also increases the processing time and makes the model more complex. Deep learning with epoch 10.0 is applied in this research study.

### Performance Evaluation

After training and constructing a prediction model, its performance is tested on similar features with an unseen output class to evaluate how accurately the model performs. A confusion matrix is derived to check the similarity between the actual output and predictions of the designed model. In the confusion matrix, true positive (TP) indicates that positive output is predicted as positive, false positive (FP) indicates that negative class is predicted as a positive class, true negative (TN) represents the true prediction of negative class, and false negative (FN) shows that a negative class is falsely predicted as the positive class. These are the primary measures for any classification model. One of the most used performance measures is accuracy. It is the ratio of the correct number of predictions to the total number of instances. Equations 1, describe the accuracy formula.


(1) $$\begin{array}{r} \text{Accuracy}=\;\frac{\text{True Positive}+\text{True Negative}}{\text{All}} \end{array}$$

### Experimental Tool and Setup

In this research study, RapidMiner is used to implement classification algorithms. RapidMiner is a user-friendly platform for the implementation of artificial intelligence algorithms to solve data analysis and data mining problems. It is open-source software with multiple license types for user convenience. However, it is freely available for academic use. RapidMiner contains hundreds of classification, regression, clustering, and feature ranking operators that are used without coding skills (94). RapidMiner is designed for data mining, data pre-processing and visualization, statistical analysis, feature extraction, prediction, and classification. RapidMiner is easy to use as it has input, output, and processing operators that need drag and drop functions and connection building simply. RapidMiner is widely used for machine learning, text mining, sentiment analysis, future predictions, and statistical data analysis. Its Java-based graphical user interface allows business persons and researchers to use it for data preparation, processing, and business future predictions (95).

### K-Fold Cross-Validation

K-fold cross-validation is a technique for dividing data into subsets for training and testing of the model. This technique is mainly used with prediction models to evaluate how the designed model will perform on new unseen data. In predictive analysis, a model is generally trained on a dataset available with the required class labels, and when the model is built, it is used for testing on a new dataset with unknown output labels, so that model performance is evaluated (15). There are two techniques for dataset splitting: split data and cross-validation. K-fold cross-validation has an advantage over the split data technique because it works efficiently when applied to a biased dataset. K represents the number of times the data is trained with k-1 subsets for training and 1 subset for testing. In cross-validation, each instance is checked for both training and testing both. The ten-fold cross-validation is used to train and test the classification models.

## Results and Discussions

**Figure 3:**
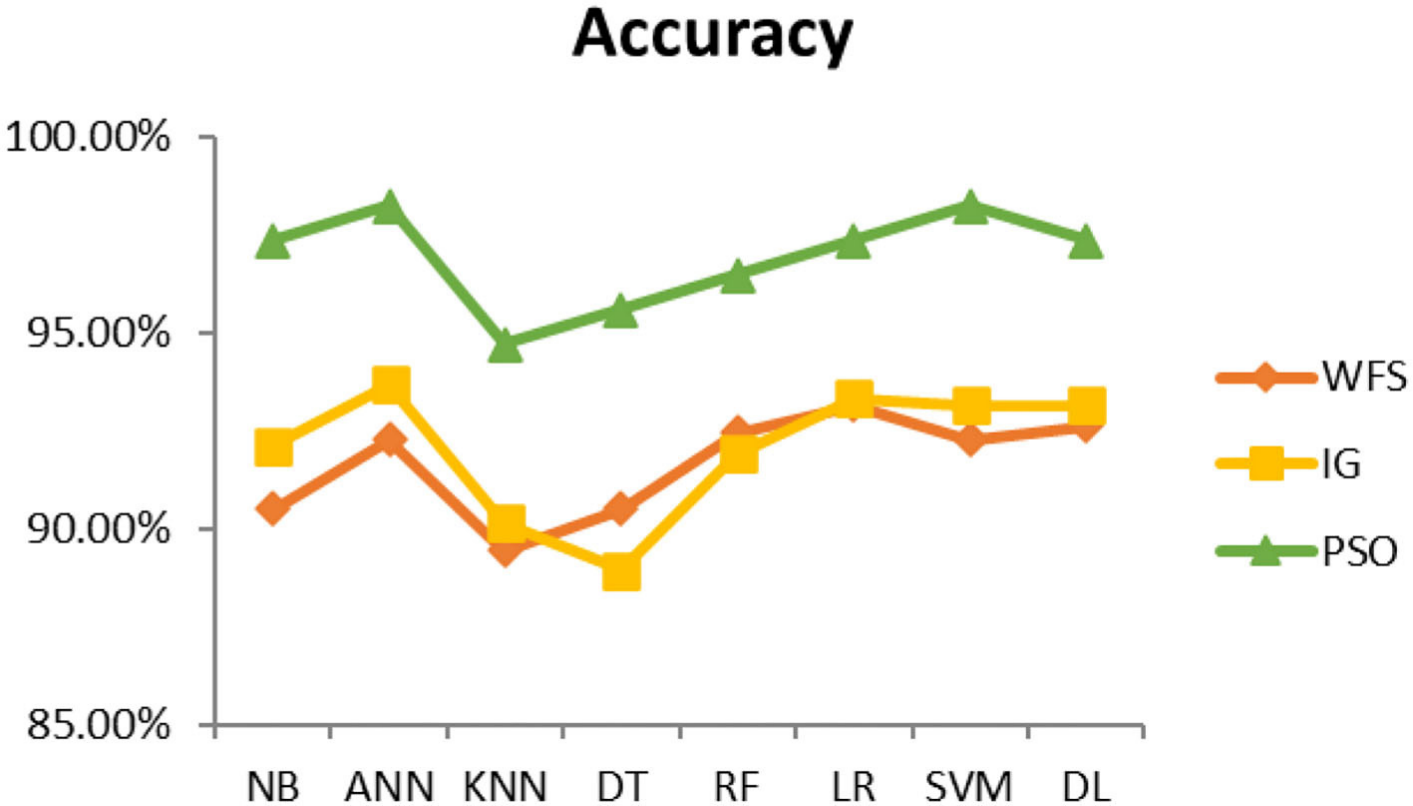
Cancer diagnosis accuracy (dataset 1).

**Figure 4:**
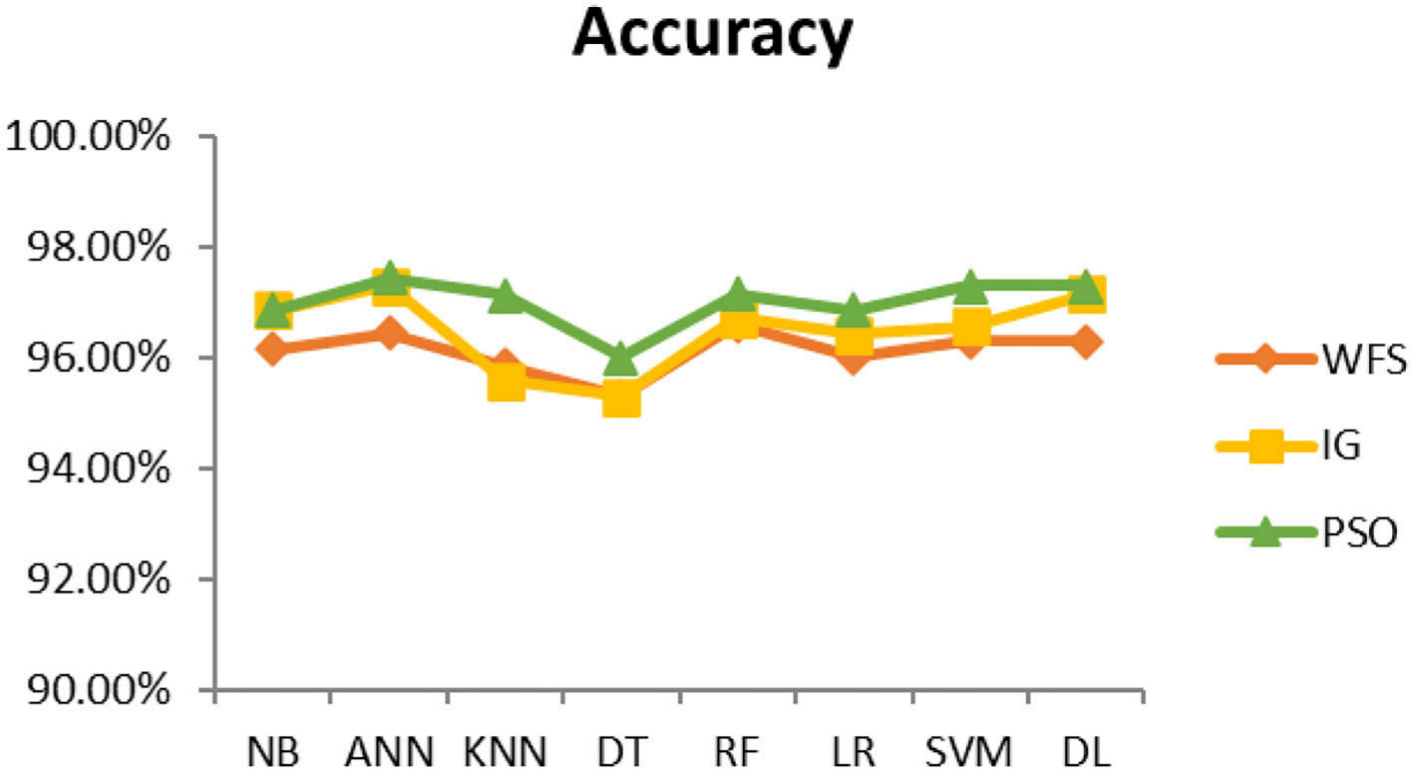
Cancer diagnosis accuracy (dataset 2).

**Figure 5:**
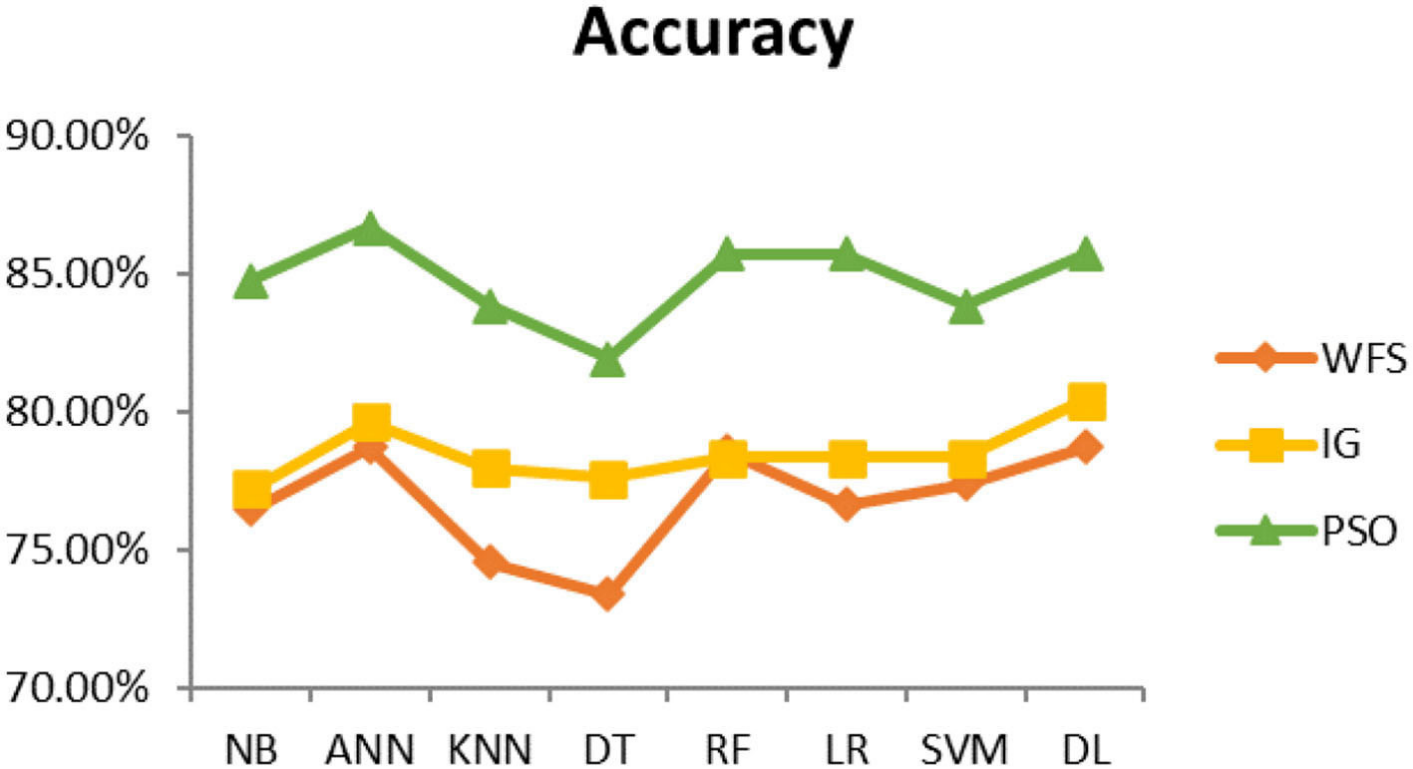
Diabetes diagnosis accuracy (dataset 1).

**Figure 6:**
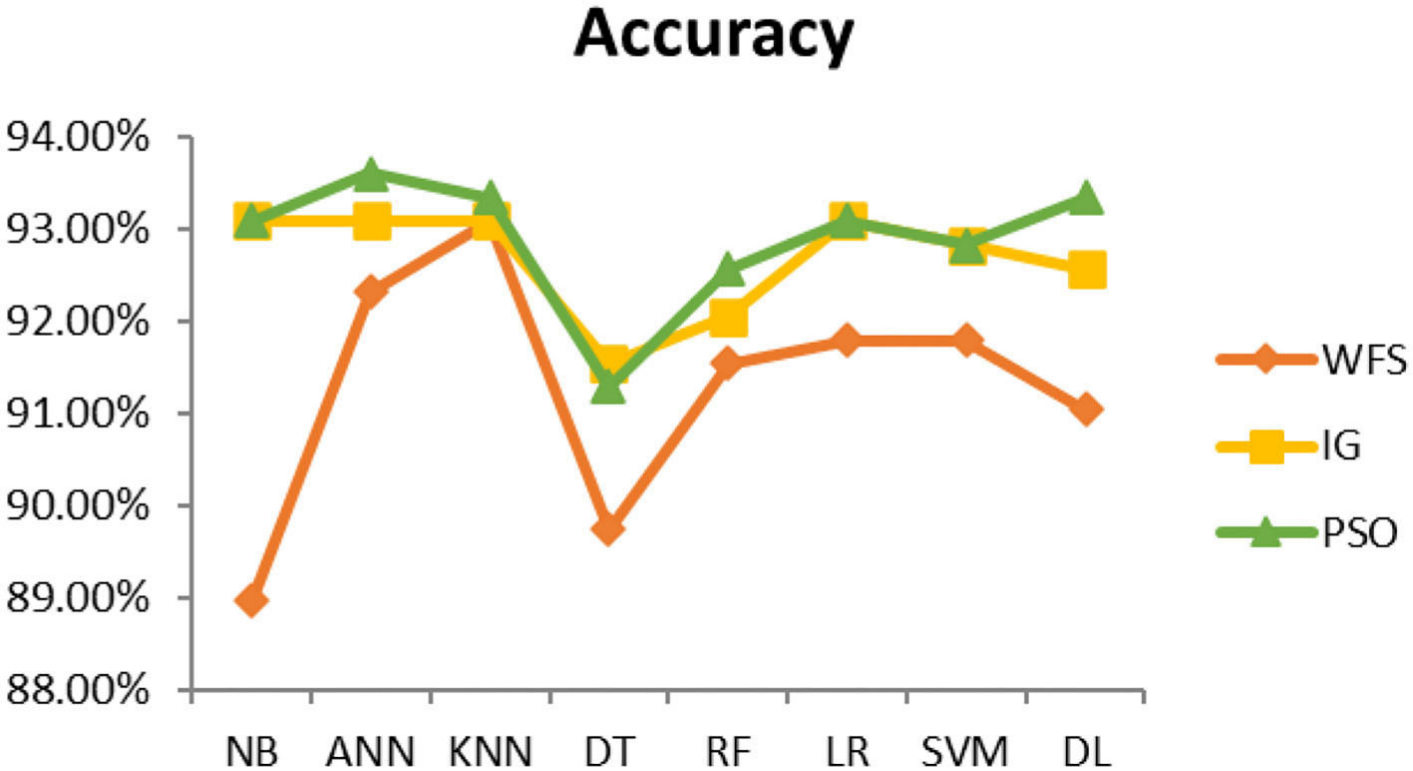
Diabetes diagnosis accuracy (dataset 2).

**Figure 7:**
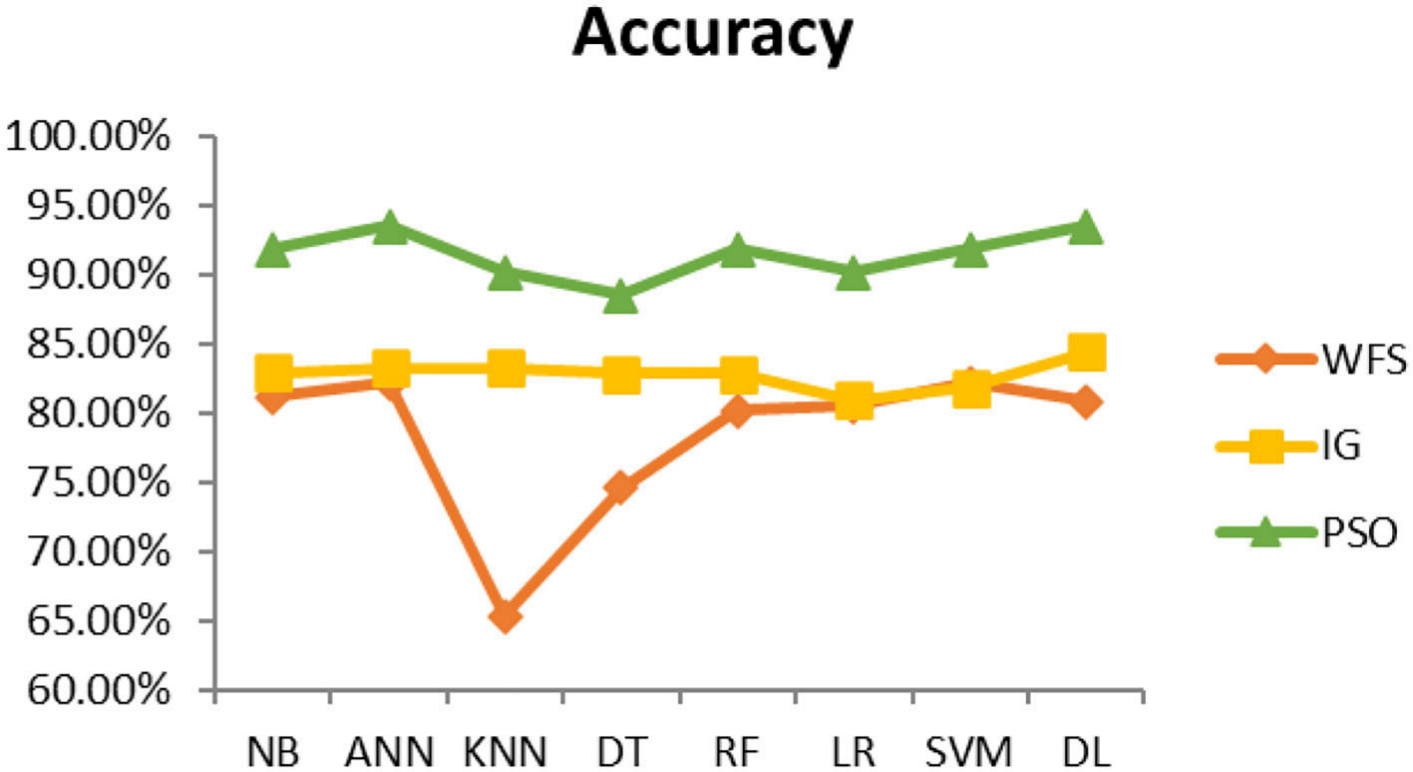
Heart attack diagnosis accuracy (dataset 1).

**Figure 8:**
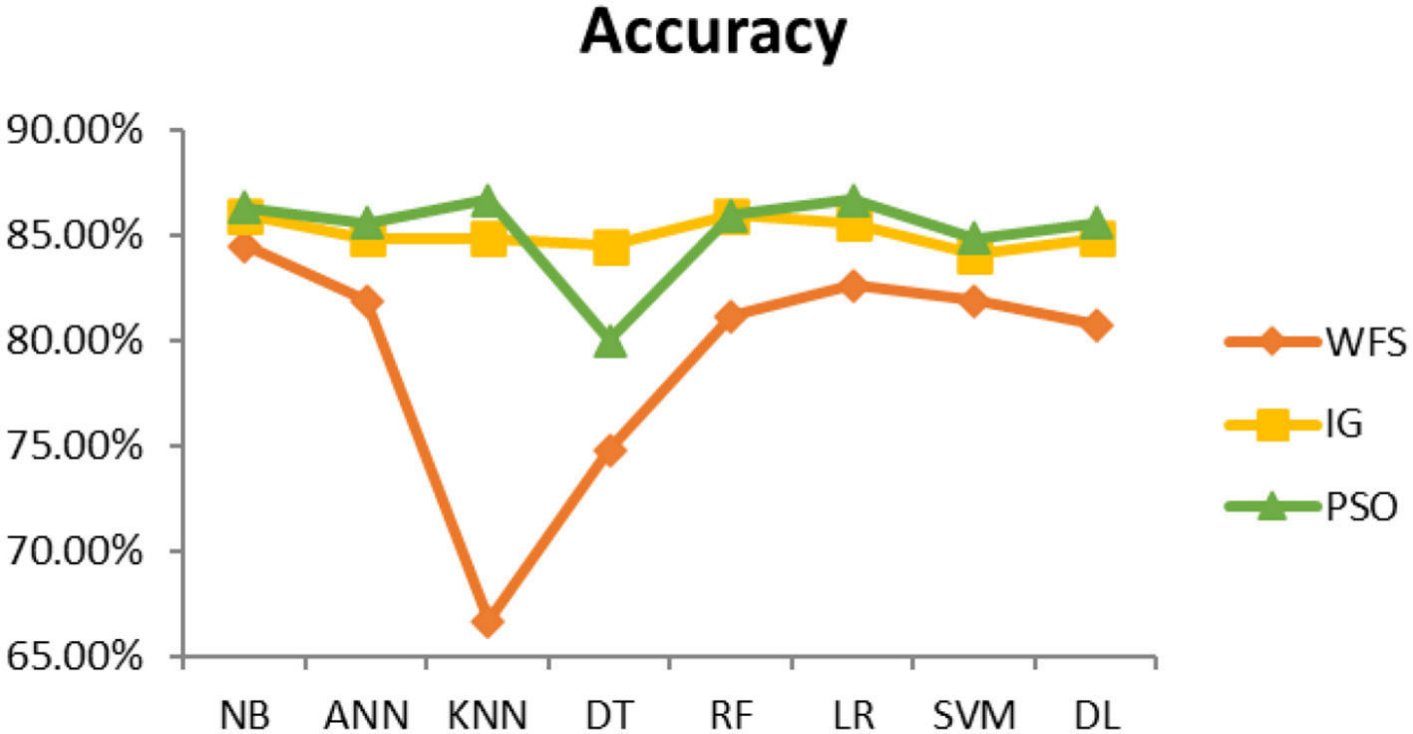
Heart attack diagnosis accuracy (dataset 2).

**Figure 9:**
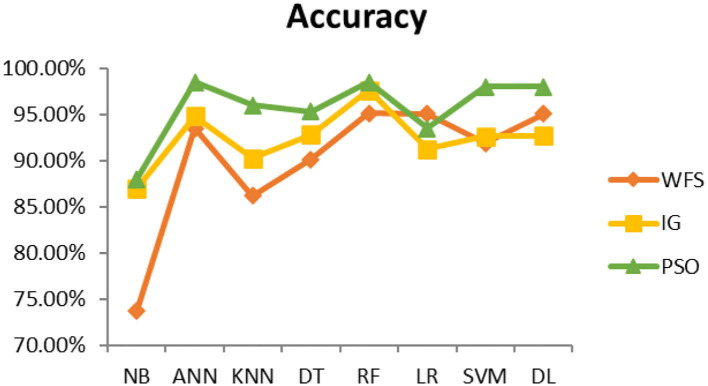
Hepatitis diagnosis accuracy (dataset 1).

**Figure 10:**
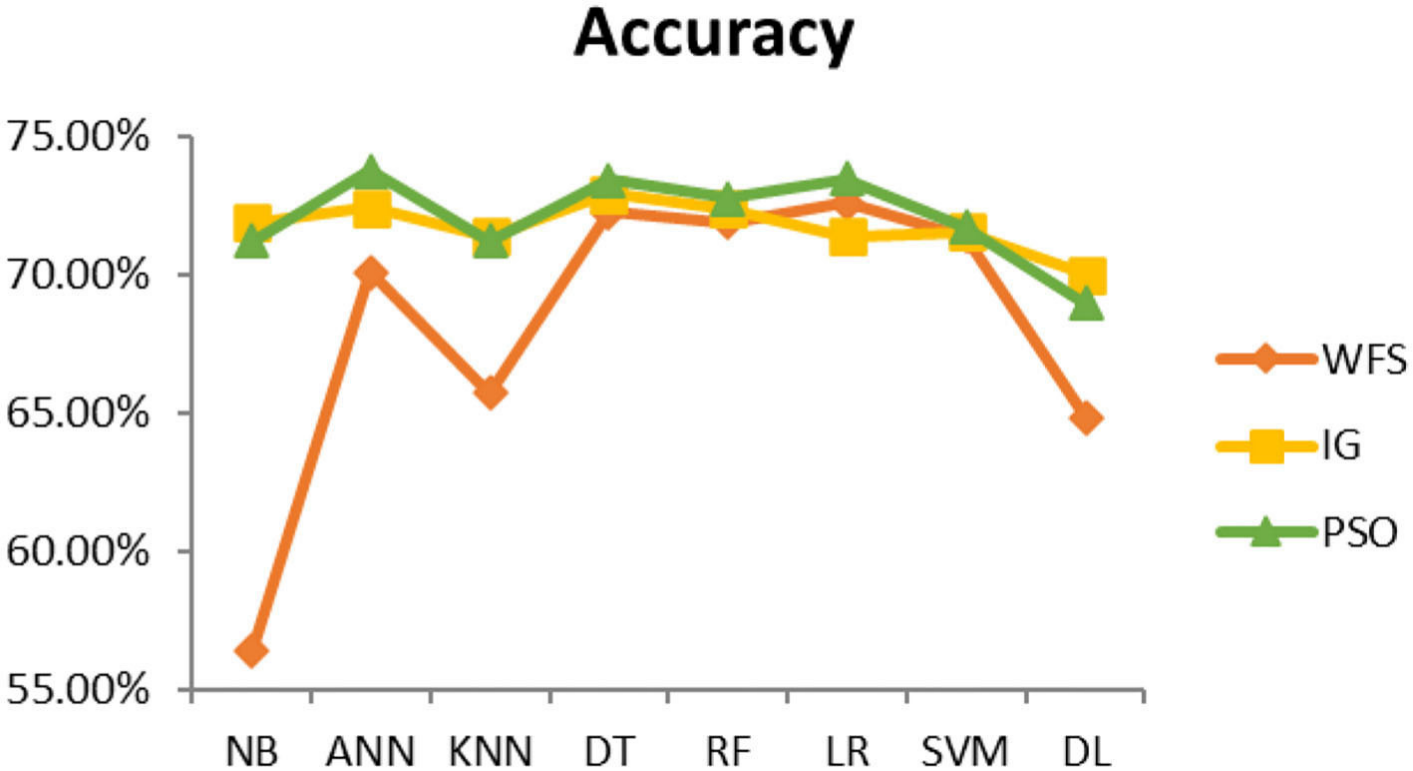
Hepatitis diagnosis accuracy (dataset 2).

**Figure 11:**
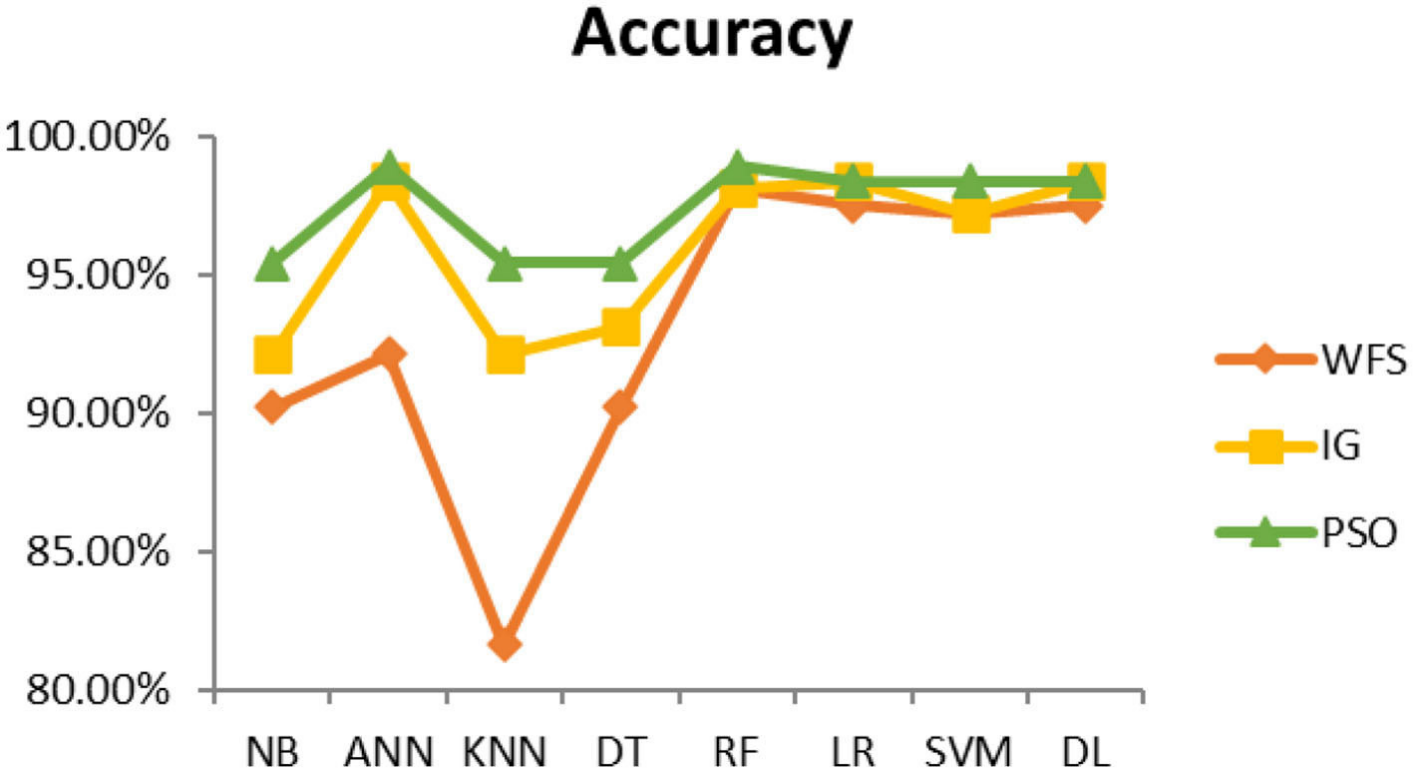
Kidney disease diagnosis accuracy.

**Figure 12:**
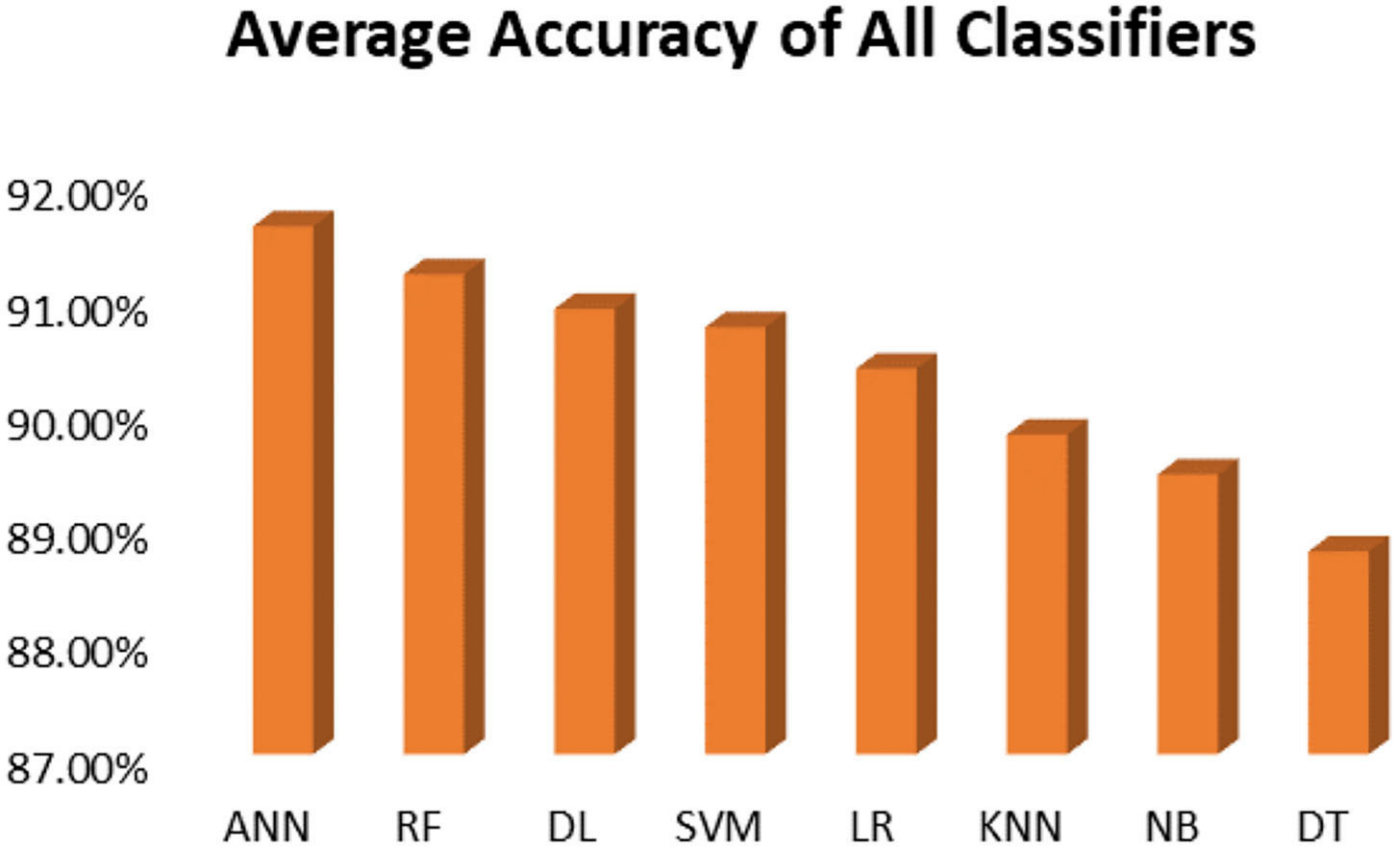
Average accuracy achieved by all classification algorithms.

This section presents the results achieved and then briefly describes these results. The ANN classification algorithm applied to feature selection using PSO. The performance of the classifiers is presented using standard measure of accuracy. Seven classification algorithms are applied to each dataset to compare their prediction performance with the proposed model. The prediction performance of ANN is improved using the PSO feature selection approach. Results with and without feature selection are compared to explore the impact of the most influential features and to present how the proposed feature optimization method performs better than other techniques. Figure 3 presents the accuracy of the first breast cancer dataset and shows that logistic regression without feature selection (WFS) gives the highest accuracy of 93.15%, while the information gain increased the performance of ANN and achieved the highest accuracy of 93.67%. Finally, PSO is applied and a 5% increase in accuracy is observed. Therefore, the proposed model obtains the highest accuracy of 98.23%. For the second breast cancer dataset, as shown in Figure 4, Random Forest without feature selection gave the highest accuracy of 96.57%, followed by ANN with an accuracy of 96.43%, which increases to 97.43% after the selection of features by PSO. For diabetes prediction, as shown in Figure 5, ANN and Deep Learning achieve the highest accuracy of 78.72%. After feature extraction with PSO, the accuracy of ANN increases to 86.67%, which is the highest compared to other classification algorithms. When the proposed model is applied to another dataset for diabetes prediction, as shown in Figure 6, the highest accuracy of 93.59% is observed in ANN applied to PSO. Therefore, for both diabetes patient datasets, the proposed model reveals the highest results. For heart attack prediction, as shown in Figure 7, ANN and Deep Learning provide the highest accuracy of 82.17 and 82.13%, respectively. The most significant features are extracted with PSO, which shows an accuracy of 93.44% obtained by the proposed model. The k-NN shows a remarkable increase with an accuracy of 68.88% without feature selection and 90.62% with PSO. The proposed model is applied to another dataset for heart attack prediction, as shown in Figure 8, and PSO is found to show a remarkable increase in accuracy. ANN gave an accuracy of 81.85%, which increases to 85.56% after selecting the most significant features with PSO. Figure 9 presents a comparison of eight classification algorithms with the proposed model for prediction of hepatitis disease. Random Forest achieved the highest accuracy of 95.13%, while ANN gave an accuracy of 93.46%. After feature selection, the proposed model obtained an accuracy of 98.46%. Next, the proposed model evaluates another dataset for hepatitis prediction in order to examine the results more accurately. Figure 10 shows that the proposed model achieved highest accuracy of 73.73%. Figure 11 shows the accuracy of kidney disease prediction. It shows that after PSO optimization, ANN and Random Forest give 98.90% accurate results. Information Gain shows a prominent increase in accuracy, but PSO significantly gives the best results. Different datasets have different accuracy because of different types of features and the number of instances. Optimization techniques help classifiers reduce irrelevant and useless features, remove outliers, and convert complex data into a simple form. It is found that overall, ANN, Random Forest, and Deep Learning give the best results, but ANN outperformed all other techniques. Table 3 presents the highest accuracy gained by the proposed model for all five selected diseases. Table 4 and Figure 12 present the overall accuracy of the eight prediction algorithms achieved with PSO feature selection. An average of the results obtained by each prediction algorithm is calculated and it is found that the proposed ANN model produces the most accurate results.

**Table 3:** Highest accuracy achieved by proposed model.

**Diseases**	**Highest results achieved by proposed model**
Cancer	98.23%
Diabetes	93.59%
Heart	93.44%
Hepatitis	98.46%
Kidney	98.90%

**Table 4:** Overall accuracy achieved for all diseases prediction.

**Model**	**Cancer**	**Cancer**	**Diabetes**	**Diabetes**	**Heart**	**Heart**	**Hepatitis**	**Hepatitis**	**Kidney**	**Average**
ANN	98.23%	97.43%	86.67%	93.08%	93.44%	85.56%	98.46%	72.73%	98.90%	91.61%
RF	96.46%	97.14%	85.71%	93.59%	91.80%	85.93%	98.46%	72.74%	98.90%	91.19%
DL	97.35%	97.28%	85.71%	93.33%	93.44%	85.56%	98.00%	68.97%	98.38%	90.89%
LR	97.35%	96.86%	85.71%	91.28%	90.16%	86.67%	93.46%	73.44%	98.38%	90.37%
SVM	98.23%	97.28%	83.81%	92.56%	91.80%	84.81%	98.00%	71.70%	98.38%	90.73%
KNN	94.69%	97.13%	83.81%	93.08%	90.16%	86.67%	96.00%	71.19%	95.40%	89.79%
NB	97.35%	96.86%	84.76%	93.33%	91.80%	86.30%	88.00%	71.20%	95.40%	89.44%
DT	95.58%	96.00%	81.90%	92.82%	88.52%	80.00%	95.30%	73.42%	95.40%	88.77%

**Figure 13:**
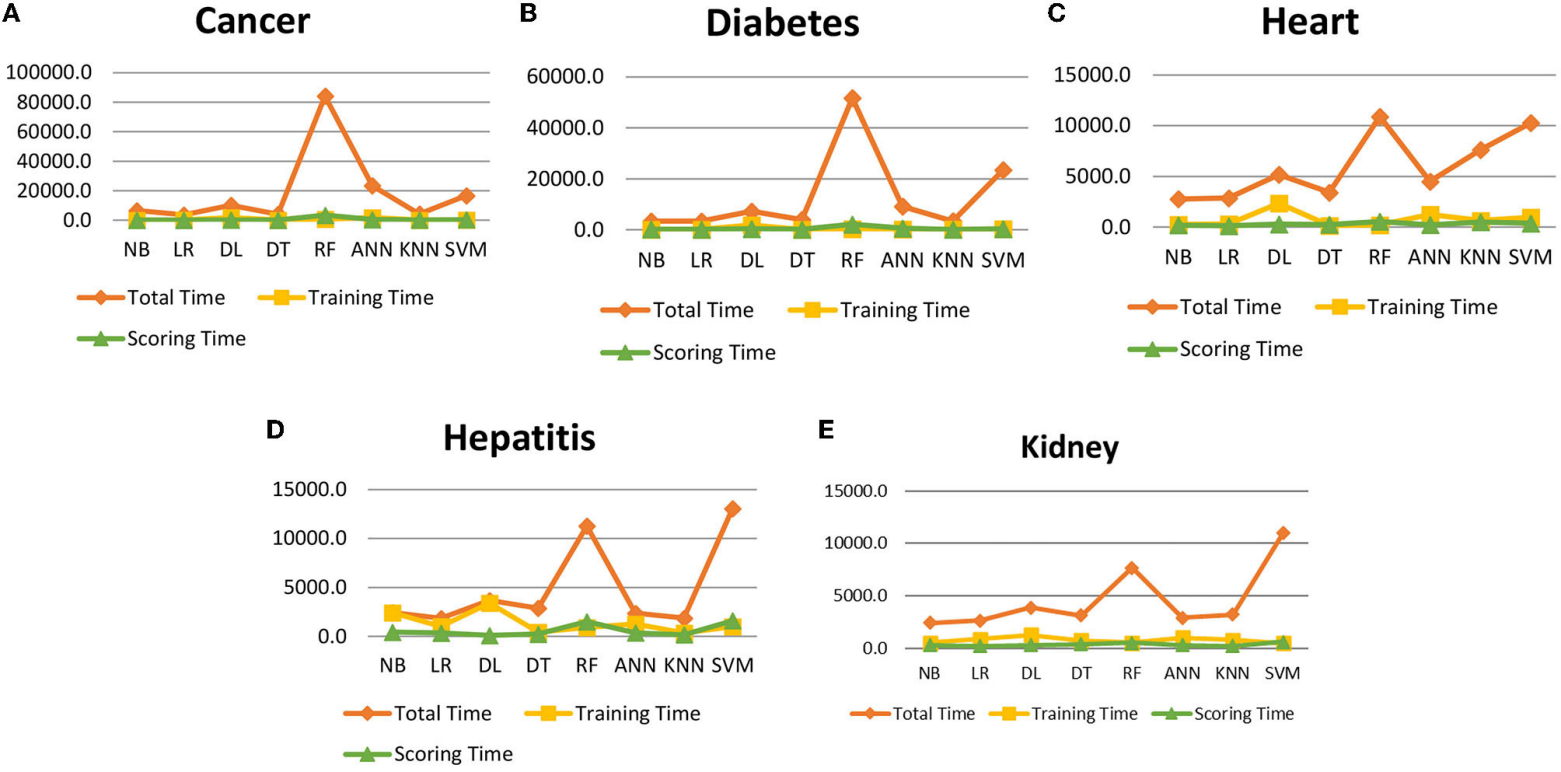
Data processing time for cancer, diabetes, heart, hepatitis and kidney disease.

It is shown that the proposed model gives the highest results, that is, 91.61%, followed by Random Forest with an accuracy of 91.19%. However, the Random Forest required more processing time compared to ANN. Therefore, the processing time of different classification techniques is also evaluated. Only one dataset for each disease is evaluated for processing time comparison. Total processing time is calculated in milliseconds (ms). Figure 13 presents the total training and testing processing time for cancer, diabetes, heart, hepatitis, and kidney. The cancer dataset shows that Random Forest takes the most time. The prediction time required for the diabetes dataset also indicates that Random Forest requires more time compared to other classification techniques. The processing time for a heart attack shows that Random Forest followed by the SVM algorithm takes additional time. Hepatitis data processing time shows that SVM followed by Random Forest requires more time for training and testing. Random Forest and SVM are shown to be the slowest prediction and data processing algorithms for kidney disease. Overall, Figure 13 shows that Random Forest and SVM required the most time for processing. Random Forest is an ensemble technique; its processing time depends on the number of trees combined to construct a forest, while SVM processing time depends on the kernel type. However, Naive Bayes and Logistic Regression take minimal time to build classification models. Furthermore, the processing time is also dependent on the number of instances in the dataset. Large datasets require more time for training and testing processes for all classification techniques. In (19), 10 different datasets are used for disease prediction with multiple feature selection approaches Information Gain, Gain Ratio, and ReliefF. Their proposed model gives the highest results of 99% when used with various subsets of patient attributes. Another study (75), suggested a feature selection method using Information Gain with different numbers of k for cross-validation to early predict kidney disease and achieved an accuracy of 81%. A hybrid classification model is proposed in (2) to diagnose three chronic diseases, including kidney disease, diabetes, and Hepatitis. The probabilistic feature selection approach is applied to a Logistic Regression classifier, and 91.6% accuracy is achieved. In another study (18), a chronic disease prediction model is built with three datasets of Breast Cancer, Diabetes and Heart attack patients. Convolutional Neural Network is applied to correlation coefficient-based attributes ranking technique. Diseases are predicted in two steps, at first with all available features and secondly with features of high significance. Table 5 gives a comparative analysis of previous research studies as compared to the proposed approach.

**Table 5:** Comparative analysis.

**References**	**Year**	**Predicted diseases**	**Classification algorithms**	**Feature selection**	**Highest results**
Alam et al. (19)	2019	Cancer, diabetes, BP, hepatitis, heart, Parkinson's & carcinoma	Random Forest	Information Gain, Gain Ratio & Relief	99%
Atallah et al. (75)	2019	Kidney transplantation	K-NN	Information Gain & Naive Bayes	81%
Hegde et al. (2)	2020	Kidney, diabetes & hepatitis	Logistic Regression	Probabilistic Feature Selection	91.6%
Sandhiya et al. (18)	2020	Breast cancer, diabetes & heart	CNN	Incremental Feature Selection	93%
Arumugam et al. (77)	2021	Diabetes and breast cancer	Decision tree	Probabilistic Feature Selection	90%
Proposed	2022	Diabetes, breast cancer, hepatitis, kidney & heart diseases	ANN	PSO	99.67%

## Conclusion

Early diagnosis of chronic diseases is a major research challenge. Various artificial intelligence techniques are used in literature for medical data classification and disease prediction. Such techniques are often applicable for selected datasets to diagnose specific disease with a limited set of attributes. In this paper, chronic diseases are predicted using an augmented artificial neural network based approach. The accuracy of the proposed model is improved using the particle swarm optimization (PSO) feature selection algorithm. This is shown to eliminate irrelevant features and assign weights to the most contributing features. Our proposed approach performs predictions more effectively and efficiently than other state-of-the-art models. Five most widespread chronic diseases are selected for diagnosis, including breast cancer, diabetes, heart attack, hepatitis, and kidney disease. Nine publicly available benchmark datasets are used in this study. The proposed model comprising an ANN classifier applied with PSO based feature selection is compared with seven state-of-the-art artificial intelligence techniques: decision tree, random forest, deep learning, naive Bayes, SVM, KNN, and logistic regression. Comparative results show that our proposed model performs the best out of all eight classification algorithms, with the highest accuracy of 99.67%. Our model obtained 98.23, 93.59, 98.46, 93.44, and 98.90% prediction accuracy for breast cancer, diabetes, hepatitis, heart, and kidney diseases. Whilst our proposed model produced the best results; the classification performance is found to depend significantly on the data features used for outcome prediction. Therefore, up to two datasets for each chronic disease are further used to apply our proposed model on different attributes. Results showed that our proposed approach outperformed other models. A comparison of processing time of classifiers showed that our optimized ANN approaches required less time compared to random forest, deep learning, and SVM. Our study can play a vital role for predicting multiple diseases using optimized ANN based prediction models in hospitals and medical labs. Further optimizing essential features can be beneficial for medical purposes to reduce patients' data attributes.

## Future Work and Limitations

In future, we plan to predict more chronic diseases with our proposed model. In particular, ensemble feature selection approaches may result in better prediction of multiple chronic diseases. Developing a system that can diagnose various diseases in patients is an open research problem and there has been little research work reported in this area. The number of patients living with more than one chronic disease is growing globally. We plan to build a prediction model based on classification and feature selection approaches to diagnose two or more chronic diseases in a person. A limitation of this study is that all classification methods are applied to selected diseases, while a dataset with different patient diseases may lead to suboptimal predictions. Although our augmented artificial intelligence based prediction model has shown promising results, there are a number of open research problems. First, generalized and globally applicable systems for disease prediction are not available. Second, prediction performance in research does not scale to real-world clinical applications due to limited data availability and different sets of attributes used in research studies. Third, real-time implementation and clinical validation of prediction models is a major challenge. Other open research areas include continuously improving prediction results based on clinical validation and diagnosing previously unknown diseases.

## Data Availability Statement

The publicly available datasets are used in this study, whose details are included in the “Materials and Methods” section of this article. Please contact the corresponding author for further requests.

## Author Contributions

JR and SB: conceptualization and methodology. JR, SB, JK, MW, AH, SJ, and RK: data curation. JR, JK, and AH: formal analysis, project administration, and writing—review and editing. JK and JR: funding acquisition. JR, SB, AH, SJ, and RK: investigation. MW, JR, and JK: resources. JR, JK, SJ, and RK: software. JR and SB: validation and writing—original draft. JR, SB, and JK: visualization. All authors contributed to the article and approved the submitted version.

## Funding

This research was partly supported by the National Research Foundation of Korea (NRF) grant funded by the Korea Government (MSIT) (No. 2021R1A4A1031509) and by Basic Science Research Program through the National Research Foundation of Korea (NRF) funded by the Ministry of Education (No. 2020R1I1A3069700).

## Conflict of Interest

The authors declare that the research was conducted in the absence of any commercial or financial relationships that could be construed as a potential conflict of interest.

## Publisher's Note

All claims expressed in this article are solely those of the authors and do not necessarily represent those of their affiliated organizations, or those of the publisher, the editors and the reviewers. Any product that may be evaluated in this article, or claim that may be made by its manufacturer, is not guaranteed or endorsed by the publisher.

## References

[1] May HT Anderson JL Muhlestein JB Knowlton KU Horne BD. Intermountain chronic disease risk score (ICHRON) validation for prediction of incident chronic disease diagnoses in an australian primary prevention population. Euro J Intern Med. (2020) 79:81–87. 10.1016/j.ejim.2020.06.009 32563688

[2] Hegde S Mundada MR. Early prediction of chronic disease using an efficient machine learning algorithm through adaptive probabilistic divergence based feature selection approach. Int J Pervasive Comput Commun. (2020) 20:145. 10.1108/IJPCC-04-2020-0018

[3] Latha CBC Jeeva SC. Improving the accuracy of prediction of heart disease risk based on ensemble classification techniques. Inform. Med. Unlocked. (2019) 16:1–9. 10.1016/j.imu.2019.100203

[4] Howard N Chouikhi N Adeel A Dial K Howard A Hussain A. BrainOS: a novel artificial brain-alike automatic machine learning framework. Front. Comput. Neurosci. (2020) 14:1–15. 10.3389/fncom.2020.00016 32194389 PMC7063840

[5] Bi X Zhao X Huang H Chen D Ma Y. Functional brain network classification for Alzheimer's disease detection with deep features and extreme learning machine. Cognit Comput. (2020) 12:513–27. 10.1007/s12559-019-09688-2

[6] Guo L. Under The background of healthy china: regulating the analysis of hybrid machine learning in sports activities to control chronic diseases. Measurement. (2020) 164:1–10. 10.1016/j.measurement.2020.107847

[7] W.H.O. NonCommunicable Diseases. (2018). Available online at: https://www.who.int/newsroom/fact-sheets/detail/noncommunicable-diseases (accessed December 12, 2021).

[8] Hemanth Reddy K Saranya G. Prediction of cardiovascular diseases in diabetic patients using machine learning techniques, in Artificial Intelligence Techniques for Advanced Computing Applications, (New York, NY: Springer), p. 299–305 (2020).

[9] W.H.O. Cardiovascular diseases (CVDs). (2016). Available online at: https://www.who.int/en/news-room/fact-sheets/detail/cardiovascular-diseases-(cvds) (accessed December 12, 2021).

[10] Diabetes - A Major Risk Factor for Kidney Disease. National Kidney Foundation. (2020). Available online at: https://www.kidney.org/atoz/content/diabetes (accessed December 12, 2021).

[11] Parisi L RaviChandran N. Evolutionary feature transformation to improve prognostic prediction of hepatitis. Knowl Based Syst. (2020) 200:1–10. 10.1016/j.knosys.2020.106012

[12] Abd El-Salam SM Ezz MM Hashem S Elakel W Salama R ElMakhzangy H. Performance of machine learning approaches on prediction of esophageal varices for Egyptian chronic hepatitis C patients. Inform. Med. Unlocked. (2019) 17:1–7. 10.1016/j.imu.2019.100267

[13] Chugh G Kumar S Singh N. Survey on machine learning and deep learning applications in breast cancer diagnosis. Cogn Computat. (2021) 13:1451–70. 10.1007/s12559-020-09813-6

[14] Raj RS Sanjay DS Kusuma M Sampath S. Comparison of support vector machine and naïve bayes classifiers for predicting diabetes, In 2019 1st International Conference on Advanced Technologies in Intelligent Control, Environment, Computing & Communication Engineering (ICATIECE). (Mew York, NY: IEEE) (2019).

[15] Aada A Tiwari S. Predicting diabetes in medical datasets using machine learning techniques. Int J Scientific Eng Res Vol. (2017) 8:257–67. Available online at: https://ijsret.com/wp-content/uploads/2019/03/IJSRET_V5_issue2_154.pdf

[16] Yuvaraj N SriPreethaa KR. Diabetes prediction in healthcare systems using machine learning algorithms on Hadoop cluster. Cluster Comput. (2019) 22:1–9. 10.1007/s10586-017-1532-x

[17] Elhoseny M Shankar K Uthayakumar J. Intelligent diagnostic prediction and classification system for chronic kidney disease. Sci Rep. (2019) 9:1–14. 10.1038/s41598-019-46074-2 31270387 PMC6610122

[18] Sandhiya S Palani U. An effective disease prediction system using incremental feature selection and temporal convolutional neural network. J Amb Intell Hum Comput. (2020) 11:5547–60. 10.1007/s12652-020-01910-6

[19] Alam MZ Rahman MS Rahman MS. A Random Forest based predictor for medical data classification using feature ranking. Inform Med Unlocked. (2019) 15:1–12. 10.1016/j.imu.2019.100180

[20] Maini E Venkateswarlu B Marwaha D Maini B. Upgrading the performance of machine learning based chronic disease prediction systems using stacked generalization technique. Int J Comput Digit Syst. (2020) 10:1–9. 10.12785/ijcds/100192

[21] Aldhyani TH Alshebami AS Alzahrani MY. Soft clustering for enhancing the diagnosis of chronic diseases over machine learning algorithms. J Healthcare Eng. (2020) 2020:1–16. 10.1155/2020/4984967 32211144 PMC7085388

[22] Kanimozhi U Ganapathy S Manjula D Kannan A. An intelligent risk prediction system for breast cancer using fuzzy temporal rules. Nat Acad Sci Lett. (2019) 42:227–32. 10.1007/s40009-018-0732-0

[23] Sinha A Sahoo B Rautaray SS Pandey M. Improved framework for breast cancer prediction using frequent itemsets mining for attributes filtering, In 2019 International Conference on Intelligent Computing Control Systems (ICCS). (2019). (New York, NY: IEEE).

[24] Liu N Qi ES Xu M Gao B Liu GQ. A novel intelligent classification model for breast cancer diagnosis. Inf Process Manag. (2019) 56:609–23. 10.1016/j.ipm.2018.10.014

[25] Islam M Haque M Iqbal H Hasan M Hasan M Kabir MN. Breast cancer prediction: a comparative study using machine learning techniques. SN Comput Sci. (2020) 1:1–14. 10.1007/s42979-020-00305-w

[26] Alickovic E Subasi A. Breast cancer diagnosis using GA feature selection and Rotation Forest. Neural Comput Appl. (2017) 28:753–63. 10.1007/s00521-015-2103-9

[27] Azar AT El-Said SA. Probabilistic neural network for breast cancer classification. Neural Comput Applicat. (2013) 23:1737–51. 10.1007/s00521-012-1134-834239550

[28] Alam TM Iqbal MA Ali Y Wahab A Ijaz S Baig TI. A model for early prediction of diabetes. Inform Med Unlocked. (2019) 16:100204. 10.1016/j.imu.2019.100204

[29] Oladele TO Ogundokun RO Kayode AA Adegun AA Adebiyi MO. Application of data mining algorithms for feature selection and prediction of diabetic retinopathy, in International Conference on Computational Science and Its Applications. (New York, NY: Springer), (2019).

[30] Gadekallu TR Khare N Bhattacharya S Singh S Maddikunta PKR Ra IH. Early detection of diabetic retinopathy using PCA-firefly based deep learning model. Electronics. (2020) 9:274. 10.3390/electronics902027434749634

[31] Gadekallu TR Khare N Bhattacharya S Singh S Maddikunta PKR Srivastava G. Deep neural networks to predict diabetic retinopathy. J Amb Intell Human Comput. (2020) 2020:1–14. 10.1007/s12652-020-01963-7

[32] Gadekallu TR Iwendi C Wei C Xin Q. Identification of malnutrition and prediction of BMI from facial images using real-time image processing and machine learning. (2021).

[33] Gadekallu TR Pham QV Huynh-The T Bhattacharya S Maddikunta PKR Liyanage M. Federated Learning for Big Data: A Survey on Opportunities, Applications, and Future Directions. arXiv preprint arXiv:2110.04160. (2021)

[34] Srinivasu PN Bhoi AK Jhaveri RH Reddy GT Bilal M. Probabilistic Deep Q Network for real-time path planning in censorious robotic procedures using force sensors. J Real-Time Image Process. (2021) 18:1773–85. 10.1007/s11554-021-01122-x

[35] Fitriyani NL Syafrudin M Alfian G Rhee J. Development of disease prediction model based on ensemble learning approach for diabetes and hypertension. IEEE Access. (2019) 7:144777–89. 10.1109/ACCESS.2019.2945129

[36] Kanwal S Rashid J Kim J Nisar MW Hussain A Batool S. An attribute weight estimation using particle swarm optimization and machine learning approaches for customer churn prediction, In: 2021 International Conference on Innovative Computing (ICIC). (2021) (Lahore: IEEE), p. 1–6.

[37] Kanwal S Rashid J Nisar MW Kim J Hussain A. An effective classification algorithm for heart disease prediction with genetic algorithm for feature selection, in 2021. Mohammad Ali Jinnah University International Conference on Computing (MAJICC). (2021) (Lahore: IEEE), p. 1–6.

[38] Nazir T Nawaz M Rashid J Mahum R Masood M Mehmood A. Detection of diabetic eye disease from retinal images using a deep learning based CenterNet model. Sensors. (2021) 21:5283. 10.3390/s21165283 34450729 PMC8398326

[39] Rashid J Shah SMA Irtaza A. An efficient topic modeling approach for text mining and information retrieval through K-means clustering. Mehran Univ Res J Eng Technol. (2020) 39:213–22. 10.22581/muet1982.2001.20

[40] Rashid J Shah SMA Irtaza A. Fuzzy topic modeling approach for text mining over short text. Inf Process Manag. (2019) 56:102060. 10.1016/j.ipm.2019.102060

[41] Rashid J Shah SMA Irtaza A Mahmood T Nisar MW Shafiq M. Topic modeling technique for text mining over biomedical text corpora through hybrid inverse documents frequency and fuzzy k-means clustering. IEEE Access. (2019) 7:146070–80. 10.1109/ACCESS.2019.2944973

[42] Rashid J Adnan Shah SM Irtaza A. A novel fuzzy k-means latent semantic analysis (FKLSA) approach for topic modeling over medical and health text corpora. J Intell Fuzzy Syst. (2019) 37:6573–88. 10.3233/JIFS-182776

[43] Rashid J Mahmood T Nisar MW Nazir T. Phishing detection using machine learning technique, In 2020 First International Conference of Smart Systems Emerging Technologies (SMARTTECH). (2020) (New York, NY: IEEE).

[44] Ashraf R Mehmood MS Mahmood T Rashid J Nisar MW Shah M. An efficient forensic approach for copy-move forgery detection via discrete wavelet transform, In 2020 International Conference on Cyber Warfare Security (ICCWS). (2020). (New York, NY: IEEE).

[45] Fatima M Nisar MW Rashid J Kim J Kamran M Hussain A. A novel fingerprinting technique for data storing and sharing through clouds. Sensors. (2021) 21:7647. 10.3390/s21227647 34833723 PMC8619563

[46] Aksa M Rashid J Nisar MW Mahmood T Kwon HY Hussain A. Bitmapaligner: bit-parallelism string matching with mapreduce and hadoop. CMC-Comput Mater Continua. (2021) 68:3931–46. 10.32604/cmc.2021.016081

[47] Iqbal H Azhar E Kanwal S Rashid J Nazir T. Prevention of Beacons Collision in IEEE 802.15. 6 under coexistence, In 2020 International Symposium on Recent Advances in Electrical Engineering & Computer Sciences (RAEE & CS). (2020). (New York, NY: IEEE).

[48] Zemmal N Azizi N Sellami M Cheriguene S Ziani A AlDwairi M. Particle swarm optimization based swarm intelligence for active learning improvement: Application on medical data classification. Cogn Computat. (2020) 12:991–1010. 10.1007/s12559-020-09739-z

[49] Upadhyay H Juneja S Juneja A Dhiman G Kautish S. Evaluation of ergonomics-related disorders in online education using fuzzy AHP. Computation Intell Neurosci. (2021) 21:4971. 10.1155/2021/2214971 34616442 PMC8490033

[50] Juneja S Juneja A Dhiman G Behl S Kautish S. An approach for thoracic syndrome classification with convolutional neural networks. Comput Math Methods Med. (2021) 21:584. 10.1155/2021/3900254 34594396 PMC8478541

[51] Juneja A Juneja S Soneja A Jain S. Real time object detection using cnn based single shot detector model. J Inform Technol Manage. (2021) 13:62–80. 10.22059/jitm.2021.80025

[52] Juneja S Jain S Suneja A Kaur G Alharbi Y Alferaidi A. Gender and age classification enabled blockschain security mechanism for assisting mobile application. IETE J Res. (2021) 2021:1–13. 10.1080/03772063.2021.1982418

[53] Samant P Agarwal R. Analysis of computational techniques for diabetes diagnosis using the combination of iris-based features and physiological parameters. Neural Comput Applicat. (2019) 31:8441–53. 10.1007/s00521-019-04551-9

[54] Wu JH Li J Wang J Zhang L Wang HD Wang GL. Risk prediction of type 2 diabetes in steel workers based on convolutional neural network. Neural Comput Applicat. (2019) 2019:1-16. 10.1007/s00521-019-04489-y

[55] Mohan S Thirumalai C Srivastava G. Effective heart disease prediction using hybrid machine learning techniques. IEEE Access. (2019) 7:81542–54. 10.1109/ACCESS.2019.2923707

[56] Khourdifi Y Bahaj M. Heart disease prediction and classification using machine learning algorithms optimized by particle swarm optimization and ant colony optimization. Int J Intell Eng Syst. (2019) 12:242–52. 10.22266/ijies2019.0228.24

[57] Rashid J Nisar MW. How to improve a software quality assurance in software development-a survey. Int J Comput Sci Inform Secur. (2016) 14:99. Available online at: https://www.academia.edu/28701432/How_to_Improve_a_Software_Quality_Assurance_in_Software_Development-A_Survey

[58] Kamran M Rashid J Nisar MW. Android fragmentation classification, causes, problems and solutions. Int J Comput Sci Inform Secur. (2016) 14:992. Available online at: https://www.academia.edu/30688611/Android_Fragmentation_Classification_Causes_Problems_and_Solutions

[59] Khan M Rashid J Nisar MW. A CMMI complaint requirement development life cycle. Int J Comput Sci Inform Secur. (2016) 14:1000. Available online at: https://www.academia.edu/30688621/A_CMMI_Complaint_Requirement_Development_Life_Cycle

[60] Rashid J Nisar MW. A study on semantic searching, semantic search engines and technologies used for semantic search engines. Int J Inform Technol Comput Sci (IJITCS). (2016) 10:82–9. 10.5815/ijitcs.2016.10.10

[61] Adnan SM Fatima T Habiba U Ilyas M Adnan SM Ahmad W. Matching based algorithm for semantic search and its implementation. Technic J. (2018) 23:70–6. Available online at: https://tj.uettaxila.edu.pk/index.php/technical-journal/article/view/596/52

[62] Tarawneh M Embarak O. Hybrid approach for heart disease prediction using data mining techniques, In International Conference on Emerging Internetworking, Data & Web Technologies. (2019) (New York, NY: Springer).

[63] Reddy GT Reddy M Lakshmanna K Rajput DS Kaluri R Srivastava G. Hybrid genetic algorithm and a fuzzy logic classifier for heart disease diagnosis. Evol Intell. (2020) 13:185–96. 10.1007/s12065-019-00327-1

[64] Batool S Rashid J Nisar MW Kim J Mahmood T Hussain A. A random forest students' performance prediction (rfspp) model based on students' demographic features, In: (2021) Mohammad Ali Jinnah University International Conference on Computing (MAJICC). New York, NY: Springer (2021).

[65] Farooq K Hussain A. A novel ontology and machine learning driven hybrid cardiovascular clinical prognosis as a complex adaptive clinical system. Complex Adapt Syst Model. (2016) 4:12. 10.1186/s40294-016-0023-x

[66] Parisi L RaviChandran N Manaog ML. A novel hybrid algorithm for aiding prediction of prognosis in patients with hepatitis. Neural Comput Applicat. (2020) 32:3839–52. 10.1007/s00521-019-04050-x

[67] Nilashi M Ahmadi H Shahmoradi L Ibrahim O Akbari E. A predictive method for hepatitis disease diagnosis using ensembles of neuro-fuzzy technique. J Infect Public Health. (2019) 12:13–20. 10.1016/j.jiph.2018.09.009 30293875

[68] Agarwal GG Singh AK Venkatesh V Wal N. Determination of risk factors for hepatitis C by the method of random forest. Annal Infect Dis Epidemiol. (2019) 4:1. Available online at: http://www.remedypublications.com/open-access/determination-of-risk-factors-for-hepatitis-c-by-the-method-of-random-forest-1797.pdf28656273

[69] Nusinovici S Tham YC Yan MYC Ting DSW Li J Sabanayagam C. Logistic regression was as good as machine learning for predicting major chronic diseases. J Clin Epidemiol. (2020) 122:56–69. 10.1016/j.jclinepi.2020.03.002 32169597

[70] Lu H Uddin S Hajati F Moni MA Khushi M. A patient network-based machine learning model for disease prediction: The case of type 2 diabetes mellitus. Appl Intell. (2021) 2021:1–12. 10.1007/s10489-021-02533-w23879411

[71] Damodara K Thakur A. Adaptive neuro fuzzy inference system based prediction of chronic kidney disease, In 2021 7th International Conference on Advanced Computing Communication Systems (ICACCS). (2021) (New York, NY: IEEE).

[72] Rady EHA Anwar AS. Prediction of kidney disease stages using data mining algorithms. Inform Me Unlocked. (2019) 15:100178. 10.1016/j.imu.2019.10017827861591

[73] Zhao J Gu S McDermaid A. Predicting outcomes of chronic kidney disease from EMR data based on Random Forest Regression. Math Biosci. (2019) 310:24–30. 10.1016/j.mbs.2019.02.001 30768948 PMC6435377

[74] Jongbo OA Adetunmbi AO Ogunrinde RB Badeji-Ajisafe B. Development of an ensemble approach to chronic kidney disease diagnosis. Scientific African. (2020) 8:e00456. 10.1016/j.sciaf.2020.e00456

[75] Atallah DM Badawy M El-Sayed A Ghoneim MA. Predicting kidney transplantation outcome based on hybrid feature selection and KNN classifier. Multimed Tools Appl. (2019) 78:20383–407. 10.1007/s11042-019-7370-5

[76] Lambert JR Arulanthu P Perumal E. Identification of nominal attributes for intelligent classification of chronic kidney disease using optimization algorithm, In. 2020 International Conference on Communication Signal Processing (ICCSP). (2020) (New York, NY: IEEE).

[77] Arumugam K Naved M Shinde PP Leiva-Chauca O Huaman-Osorio A. Multiple disease prediction using Machine learning algorithms. Materials Today. (2021) 7:361. 10.1016/j.matpr.2021.07.361

[78] Breast Cancer Wisconsin (Diagnostic) Data Set. Available Online At: https://www.kaggle.com/uciml/breast-cancer-wisconsin-data (accessed 26-10-2020).

[79] Breast Cancer Wisconsin. Available Online At: https://github.com/zahangirbd/medical_data_for_classification/tree/master/data/Breast%20Cancer%20Wisconsin (accessed 26-10-2020).

[80] Pima Indians Diabetes Database. Available Online at: https://www.kaggle.com/uciml/pima-indians-diabetes-database (accessed 26-10-2020).

[81] Diabetes Classification. Available Online at: https://data.world/informatics-edu/diabetes-prediction (accessed 25-10-2020).

[82] Heart Disease UCI. Available Online at: https://www.kaggle.com/ronitf/heart-disease-uci (accessed 25-10-2020).

[83] Heart Disease Prediction. Available online at: https://data.world/informatics-edu/heart-disease-prediction (accessed 26-10-2020).

[84] Hepatitis. Available online at: https://www.kaggle.com/harinir/hepatitis (accessed 26-10-2020).

[85] Indian Liver Patient Records. Available online at: https://www.kaggle.com/uciml/indian-liver-patient-records (accessed 27-10-2020).

[86] Kidney Disease Dataset. Available online at: https://www.kaggle.com/akshayksingh/kidney-disease-dataset (accessed 26-10-2020.)

[87] Zhu C Idemudia CU Feng W. Improved logistic regression model for diabetes prediction by integrating PCA and K-means techniques. Inform Med Unlocked. (2019) 17:100179. 10.1016/j.imu.2019.100179

[88] Joloudari JH Saadatfar H Dehzangi A Shamshirband S. Computer-aided decision-making for predicting liver disease using PSO-based optimized SVM with feature selection. Inform Med Unlocked. (2019) 17:100255. 10.1016/j.imu.2019.100255

[89] Mujumdar A Vaidehi V. Diabetes prediction using machine learning algorithms. Procedia Comput Sci. (2019) 165:292–9. 10.1016/j.procs.2020.01.04735046014

[90] Usama M Qadir J Raza A Arif H Yau KLA Elkhatib Y. Unsupervised machine learning for networking: Techniques, applications and research challenges. IEEE Access. (2019) 7:65579–615. 10.1109/ACCESS.2019.291664834837860

[91] Uddin S Khan A Hossain ME Moni MA. Comparing different supervised machine learning algorithms for disease prediction. BMC Med Inform Decis Mak. (2019) 19:1–16. 10.1186/s12911-019-1004-8 31864346 PMC6925840

[92] Jo T Nho K Saykin AJ. Deep learning in Alzheimer's disease: diagnostic classification and prognostic prediction using neuroimaging data. Front Aging Neurosci. (2019) 11:220. 10.3389/fnagi.2019.00220 31481890 PMC6710444

[93] LaPierre N Ju CJT Zhou G Wang W. MetaPheno: A critical evaluation of deep learning and machine learning in metagenome-based disease prediction. Methods. (2019) 166:74–82. 10.1016/j.ymeth.2019.03.003 30885720 PMC6708502

[94] Mohammed AB. Decision tree, naïve bayes and support vector machine applying on social media usage in NYC/comparative analysis. Tikrit J Pure Sci. (2018) 22:94–9. Available online at: https://www.iasj.net/iasj/download/7fe80bcbbfee924d

[95] Arunadevi DJ Ramya S Raja MR. A study of classification algorithms using Rapidminer. Int J Pure Appl Math. (2018) 119:15977–88. Available online at: https://www.acadpubl.eu/hub/2018-119-12/articles/6/1478.pdf

